# STOML2 restricts mitophagy and increases chemosensitivity in pancreatic cancer through stabilizing PARL-induced PINK1 degradation

**DOI:** 10.1038/s41419-023-05711-5

**Published:** 2023-03-11

**Authors:** Cheng Qin, Yuanyang Wang, Bangbo Zhao, Zeru Li, Tianyu Li, Xiaoying Yang, Yutong Zhao, Weibin Wang

**Affiliations:** 1 Department of General Surgery, Peking Union Medical College Hospital, Peking Union Medical College, Chinese Academy of Medical Sciences, 100023 Beijing, P.R. China; 2 Key Laboratory of Research in Pancreatic Tumor, Chinese Academy of Medical Sciences, 100023 Beijing, P.R. China; 3 National Science and Technology Key Infrastructure on Translational Medicine in Peking Union Medical College Hospital, 100023 Beijing, P.R. China

**Keywords:** Gastrointestinal cancer, Mitophagy

## Abstract

Pancreatic cancer remains one of the most lethal diseases with a relatively low 5-year survival rate, and gemcitabine-based chemoresistance occurs constantly. Mitochondria, as the power factory in cancer cells, are involved in the process of chemoresistance. The dynamic balance of mitochondria is under the control of mitophagy. Stomatin-like protein 2 (STOML2) is located in the mitochondrial inner membrane and is highly expressed in cancer cells. In this study, using a tissue microarray (TMA), we found that high STOML2 expression was correlated with higher survival of patients with pancreatic cancer. Meanwhile, the proliferation and chemoresistance of pancreatic cancer cells could be retarded by STOML2. In addition, we found that STOML2 was positively related to mitochondrial mass and negatively related to mitophagy in pancreatic cancer cells. STOML2 stabilized PARL and further prevented gemcitabine-induced PINK1-dependent mitophagy. We also generated subcutaneous xenografts to verify the enhancement of gemcitabine therapy induced by STOML2. These findings suggested that STOML2 regulated the mitophagy process through the PARL/PINK1 pathway, thereby reducing the chemoresistance of pancreatic cancer. STOML2-overexpression targeted therapy might be helpful for gemcitabine sensitization in the future.

## Introduction

Pancreatic ductal adenocarcinoma, generally referring to pancreatic cancer, remains one of the most lethal diseases. Compared with the obvious progress of survival benefits in many other cancer types, the current 5-year survival rate of pancreatic cancer is still approximately 11% [1]. Gemcitabine (GEM)-based regimens remain first-line chemotherapies. However, chemoresistance frequently occurs and significantly threatens the long-term survival of pancreatic cancer patients. In a variety of chemoresistance mechanisms, metabolic alterations, such as glycolysis, have long been widely studied [2]. In addition to enhanced glycolysis in pancreatic cancer, some mitochondrial and key subcellular compartments in energy production are also vital for pancreatic cancer progression by regulating tumor anabolism, calcium homeostasis and redox balance [3]. Recently, more evidence has indicated that mitochondria participate in the process of chemoresistance [4].

Normal and healthy mitochondria are “power factories” in cancer cells, which serve as the location for the tricarboxylic acid cycle and generate adenosine triphosphate (ATP). The quantity and quality of mitochondria are under a dynamic quality control system named mitophagy, which can selectively remove redundant mitochondria and maintain cell homeostasis [4]. Mitophagy contains classical and nonclassical pathways [5]. The classical pathway is mainly induced by PINK1 (PTEN-induced kinase 1) and PRKN (parkin RBR E3 ubiquitin protein ligase). Mechanistically, mitochondria accumulate full-length PINK1 on their outer membrane, recruiting free PRKN in the cytoplasm and resulting in the subsequent aggregation of phosphorylated Ub chains on the mitochondrial outer membrane [6]. There are many proteins that could influence the PINK1-PRKN pathway and affect mitophagy, including PARL (presenilin associated rhomboid like), a kind of intramembrane protease that is located at the mitochondrial inner membrane and can slice full-length PINK1 to cleaved PINK1. Cleaved PINK1 is degraded by the proteasome and loses its function in inducing mitophagy [7]. Previous studies have suggested that the quantity and quality of mitochondria are closely connected with cell apoptosis, which might contribute to chemoresistance [4]. Upon loss of transmembrane potential, mitochondria release proapoptotic proteins such as cytochrome C to induce a caspase cascade. In addition, mitochondria serve as the appropriate site for aerobic glycolysis, glutamine metabolism and ROS metabolism, all of which have been proven to exert a significant effect on chemoresistance [2, 4]. Therefore, mitochondria play a vital role in pancreatic cancer chemoresistance, which deserves further research.

Stomatin-like protein 2 (STOML2 or SLP2) was primarily identified as a protein located in the mitochondrial inner membrane that maintains the stability of mitochondria [8, 9]. Previous studies have suggested that STOML2 promotes the progression of multiple malignant tumors, including liver cancer [10], head and neck squamous cell carcinoma [11], ovarian cancer [12], colorectal cancer [13], and even pancreatic cancer [14]. A relevant study in pancreatic cancer carried out by Chao et al. demonstrated the relevance of STOML2 and pancreatic cancer patient prognosis. Therefore, the function and mechanism of STOML2 in pancreatic cancer deserve further study. In this study, we found that in our cohort, high STOML2 expression was related to good prognosis and was mainly located in the mitochondria, which could prevent mitophagy by stabilizing PARL and ultimately reduce chemoresistance to GEM.

## Materials and methods

### Bioinformatics analysis

The GEPIA website containing the TCGA and GTEx databases was employed. In the TCGA database, samples with high STOML2 expression (above the mean) were allocated to the high STOML2 expression group, while samples with low STOML2 expression (below the mean) were allocated to the low STOML2 expression group. The log-rank test was utilized to analyse the survival data of pancreatic cancer samples. Public proteogenomic data of pancreatic cancer tissue and Pearson correlation coefficients were employed to analyse the correlation between STOML2 and PARL at the protein level [15].

### Tissue microarray analysis and immunohistochemistry (IHC)

Tissue microarray sections (4 µm thick), including 79 pairs of pancreatic ductal adenocarcinoma samples and adjacent healthy pancreatic tissues, were purchased from Shanghai Outdo Biotech Co., Ltd. Enrolled patients received little preoperative treatment in this study. The patients involved have signed the informed consent form and the study was approved by Shanghai Outdo Biotech Co., Ltd. For immunohistochemistry (IHC), the tissue microarray section was incubated with STOML2 monoclonal antibody (1:500 dilution, Proteintech, China) overnight at 4 °C. IHC was performed according to a previously described protocol [16]. The staining results were photographed by microscopy (Nikon ECLIPSETs2R). The results of staining were independently determined by two experienced pathologists. Staining score = tumor cell proportion × staining intensity. Proportion: 0 (no stained tumor cells), 1 (<10% stained tumor cells), 2 (10–25% stained tumor cells), 3 (26–49% stained tumor cells), and 4 (≥50% stained tumor cells). Intensity: 0 (negative particles), 1 (lightly yellow particles), 2 (brownish-yellow particles) and 3 (brown particles). A staining score between 0 and 5 was defined as the low STOML2 expression group, while a score ≥ 6 was defined as the high expression group.

### Cell lines and culture conditions

The pancreatic cancer cell lines PANC1, BxPC-3, and MIA PaCa-2 were purchased from the American Type Culture Collection (ATCC, USA). PATU8988T cells were obtained from Procell Life Science & Technology Co., Ltd. (Wuhan, Hubei, China). The cells were cultured in DMEM (HyClone, Utah, USA) and RPMI-1640 (HyClone, Utah, USA) media with 10% fetal bovine serum (FBS, Biological Industries, Israel) and 1% penicillin & streptomycin (Biological Industries, Israel) and were grown in a cell incubator (37 °C with 5% CO_2_).

### Transfection assay and stable cell line construction

When seeded cells reached 70% confluence, they were infected with a STOML2-overexpression plasmid (STOML2 OE), PARL-overexpression plasmid (PARL OE) or negative control (Vector) (Shanghai Genechem, China). STOML2 cDNA or PARL cDNA was subcloned into a pcDNA3.1(+) vector. Small interfering RNA (siSTOML2, siPARL, siPINK1) and scrambled siRNA (negative control, NC) were purchased from RiboBio (Guangzhou, China) to construct knockdown cells. The transfection process was performed by Lipofectamine 3000 (Invitrogen, Lithuania) according to the manufacturer’s instructions. To construct a stable STOML2-overexpressing cell line, STOML2 (Homo sapiens) ORF sequences were cloned into Ubi-MCS-3FLAG-SV40-Puro lentiviral vectors (Shanghai Genechem, China). The lentiviral vectors were utilized to package viral particles. PANC1 cells were infected with the lentivirus for over 24 h. Then, puromycin (1 μg/ml, Sigma‒Aldrich, Missouri, USA) was added to the medium to remove uninfected cells.

### Conditional culture

At 48 h after transfection, the cells were cultured with GEM (10 μM for PANC1; 1 μM for BxPC3, Vianex S.A.-Plant C, Greece), carbonyl cyanide 3-chlorophenylhydrazone (CCCP, 10 μM, MedChemExpress, USA) or chloroquine (CQ, 10 μM, MedChemExpress, USA) for 24 h to analyse cell activities. Equal volumes of PBS or DMSO were used as controls.

### RNA isolation and qRT‒PCR

TRIzol reagent (Invitrogen, California, USA) was utilized to extract total RNA. The quantity of extracted RNA was analysed by a NanoDrop ND-1000 spectrophotometer (NanoDrop Technologies, USA). Complementary DNA was synthesized by a reverse transcription kit (ThermoFisher, Lithuania). Quantitative RT–PCR (qRT‒PCR) was conducted with SYBR Green Master Mix (Applied Biosystems, Lithuania). β-actin was used as the internal control for mRNA detection. The relative expression of mRNAs was calculated via the 2^-ΔΔCT^ method. All primers used in this study are displayed in Supplementary Table 1.

### Immunofluorescence assay

At 24 h after transfection, PANC1 or BxPC3 cells were seeded in cell culture slides (Solarbio, China). After 24 h of culture, 4% paraformaldehyde was added to the cell culture slides for 10 min at room temperature. Then, the cell culture slides were permeabilized with 1% Triton X-100 for 5 min at room temperature. Blocking was performed by 3% BSA and then incubated with anti-TOM20 at 4 °C overnight. Then, the cell culture slides were incubated with fluorophore-conjugated secondary antibodies for 1.5 h at room temperature in the dark. Finally, cell culture slides were mounted with fluorescent mounting medium containing DAPI (ZSGB-BIO, ZB-2301, China). The images were acquired by confocal microscopy (AX, Nikon). Mitochondrial number, size, and mass were analysed via ImageJ V1.53.

### Measurement of mitochondrial oxidative respiration

Seahorse assay was performed to detect mitochondrial respiratory function. After transfection with siRNA, pancreatic cancer cells (PANC1 and BxPC3, 10000 cells per well) were seeded on seahorse cell culture plates (Agilent Technologies, Seahorse XF96 V3 PS Cell Culture Microplates). The XF Calibrant was prepared for calibration of the Seahorse XFe96 Analyser. On the next day, the cells were incubated for 1 h at 37 °C without CO_2_. After calibrating the Seahorse XFe96 Analyser, the oxygen consumption rate (OCR) (performed by Agilent Technologies, Seahorse XF Cell Mito Stress Test Kit) was automatically analysed by a Seahorse XFe96 Analyser following the manufacturer’s instructions using oligomycin (1.5 μM), FCCP (2 μM), and rotenone (0.5 μM). The cells were then stained with Hoechst 33342 and counted by a Cytation™ 7 Cell Imaging Multi-Mode Reader. The OCR results were standardized by cell counts.

### Western blot and coimmunoprecipitation (Co-IP)

Proteins in cells and tissue samples were extracted by RIPA lysis buffer with protease inhibitor (Beyotime, China). Cytosolic and mitochondrial proteins were extracted using a mitochondrial extraction kit (Solarbio, China) according to the manufacturer’s instructions. The concentrations of extracted protein were quantified by a BCA Protein Assay Kit (Beyotime, China). Western blotting was performed according to previously described protocols [17]. The primary antibodies used in this study were anti-STOML2 (10348-1-AP, Proteintech, China), anti-PARL (sc-514836, Santa Cruz, Texas, USA), anti-PINK1 (6946, Cell Signaling Technology, Massachusetts, USA), anti-PINK1 (sc-518052, Santa Cruz, Texas, USA), anti-TOM20 (11802-1-AP, Proteintech, China), anti-LC3B (18725-1-AP, Proteintech, China), anti-Vinculin (66305-1-Ig, Proteintech, China), anti-Caspase-3 (sc-7272, Santa Cruz, Texas, USA), anti-Cleaved Caspase-3 (ab2302, Abcam, UK), anti-Cleaved Caspase-3 (9664, Cell Signaling Technology, Massachusetts, USA), anti-COXIV (11242-1-AP, Proteintech, China), and Anti-Ki67 (27309-1-AP, Proteintech, China). The secondary antibodies were goat anti-rabbit IgG (ZSGB-BIO, ZB-2301, China), goat anti-mouse IgG (ZSGB-BIO, ZB-2305, China), and CoraLite488-conjugated goat anti-rabbit IgG (H + L) (SA00013-2, Proteintech, China). Western blot was quantitatively analysed by ImageJ software. Fold changes under individual blots indicated the ratio to relevant control (numbers in italic and bold font). For the coimmunoprecipitation (Co-IP) assay, all procedures were accomplished according to the manufacturer’s instructions (Thermo Fisher, Massachusetts, USA). Briefly, PANC1 and BxPC3 cells were lysed in modified RIPA buffer. Cell lysates were mixed with protein A/G agarose bead-conjugated STOML2 antibody or IgG for 2 h at room temperature on a rotating plate. Then, Western blotting was performed with the primary antibody and secondary antibody (ab131366, Abcam, UK).

### Cell viability and colony formation assays

At 24 h after transfection, PANC1 or BxPC3 cells were seeded in 96-well plates (3000 cells per well). Then, cell viability was measured at 0 h, 24 h, 48 h, 72 h and 96 h using the sulforhodamine B (SRB) assay (Sigma‒Aldrich, Missouri, USA) [18]. The optical density at a wavelength of 564 nm (OD540) was measured by a microplate reader. In addition, the CCK8 assay (Dojindo Molecular Technologies, Japan) was also employed to analyse cell viability (OD450-OD630). For the colony formation assay, transfected cells were suspended at the single-cell level and plated into 6-well plates (1000 cells per well). After 14 days, the cells were stained with 0.4% crystal violet (Sigma‒Aldrich, Missouri, USA) in methanol. The plate was washed three times with water and photographed.

### Growth inhibition assay with GEM

For growth inhibition assays, pancreatic cancer cells were seeded into 96-well plates (3000 cells per well) at 24 h after transfection. After cell adherence (incubation for 4–6 h), GEM (Vianex S.A.-Plant C, Greece) concentration gradients (1 nM to 1 mM for BxPC3; 10 nM to 10 mM for PANC1) were added into each well. The SRB assay was performed after an additional 48 h of incubation to determine the cell viability and inhibition rate. The growth rate (GR) inhibition metrics were calculated according to a previously described protocol [19], which could eliminate confounders such as the cell proliferation rate and is more appropriate for measuring cellular sensitivity to GEM. The GR value is $GR(c) = 2^{\log _2(\frac{{x\left( c \right)}}{{x0}})/\log _2(\frac{{x\left( 0 \right)}}{{x0}})} - 1$. Among them, *x*0 represents the cell numbers at the start of GEM treatment, *x*(*c*) represents the number of cells in GEM-treated (concentration *c*) wells after 48 h, and *x*(*0*) represents the number of cells in control (untreated) wells at the end of the assay. GR50 is the GEM concentration at which *GR*(*c*) = 0.5. Therefore, cells with higher GR50 are more chemoresistant.

### Apoptosis assay

PANC1 and BxPC3 cells were seeded into 6-well plates. At 24 h after transfection (siRNA and plasmids), cells were treated with GEM (10 μM for PANC1; 1 μM for BxPC3, Vianex S.A.-Plant C, Greece). At 48 h after treatment, the cells were resuspended in binding buffer. Next, the cells were stained with propidium iodide (PI) and Annexin V-FITC based on the manufacturer’s instructions (Yishan Biotechnology, China). Follow-up analysis was carried out via FlowJo V10.8.1.

### Quantification of functional mitochondrial mass and reactive oxygen species (ROS)

Functional mitochondrial mass within PANC1 and BxPC3 cells was assessed by incubating cells with MitoTracker (Beyotime, China) for 30 min at 37 °C. Then, the cells were treated with DAPI (Beyotime, China) for 5 min at 37 °C. Fluorescence results for mitochondrial mass were photographed via confocal microscopy (AX, Nikon). The mitochondrial mass was finally analysed via ImageJ V1.53. For measurement of intracellular ROS levels, an ROS assay kit (Solarbio, China) was employed to measure the intracellular ROS levels of PANC1 and BxPC3 cells. Briefly, transfected PANC1 and BxPC3 cells were treated with 10 μM DCFH-DA in serum-free medium for 20 min at 37 °C. To stain the nuclei, cells were further treated with Hoechst (Beyotime, China) for 5 min at 37 °C. Fluorescence results were taken through photographs via confocal microscopy (AX, Nikon). Fluorescence results for ROS were taken through photographs via microscopy (EVOS, ThermoFisher, Massachusetts, USA). The ROS levels were finally analysed via ImageJ V1.53.

### Mitophagy detection

At 48 h after transfection, PANC1 and BxPC3 cells were seeded into cell culture imaging slides (Thermo Fisher, Massachusetts, USA) (5000 cells per chamber). After incubation for 24 h, a Mitophagy Detection Kit (Dojindo Molecular Technologies, Japan) containing mitophagy dye (red) and lysosome dye (green) was employed to assess mitophagy levels according to the manufacturer’s protocol. Mitophagy dye can be immobilized to mitochondrial proteins via thiol groups, and it can sense mitochondrial acidification during mitophagy. Additionally, lysosome dye can label lysosomes with green fluorescence [20]. Therefore, the colocalization between red and green represented the occurrence of mitophagy (yellow). Fluorescence images were taken via confocal microscopy (AX, Nikon). The mitophagy levels were finally analysed via CellProfiler V4.2.1.

### Animal experiments

All animal experiments in this study were performed under the guidelines of the Institutional Animal Care and Use Committee (Beijing, China). Control and STOML2-overexpressing PANC-1 cells were stably constructed via the aforementioned lentivirus. The cells were subcutaneously injected into the right back of 6-week-old female BALB/c nude mice (Chinese Academy of Sciences, China) (5 × 10^6^ cells in 300 μl of PBS per mouse). The minimum number of each group was 6 according to the previous publications. The mice were randomly divided into two groups, six in each group. The person who measured the tumor size was unaware to the allocation. The Tumor sizes were assessed using a calliper to measure the two perpendicular diameters of the xenografts (tumor volume (mm^3^) = 1/2 × length × width^2^). To confirm the role of STOML2 in promoting chemosensitivity in vivo, mice were administered GEM through intraperitoneal injection (25 mg/kg, twice weekly) after all of the individual tumor volume reached 80 mm^3^. After 30 days of administration, mice were euthanized. The xenografts were excised to perform further study in weight, morphology and immunohistochemistry. Ki67 and cleaved-caspase 3 expression were evaluated by IHC to illustrate the cell proliferation and cell death.

### Statistical analysis

All statistical results and graph representations were performed using GraphPad Prism 9 Software. The differences among different groups were tested using the paired or unpaired Student’s *t* test. The Kaplan‒Meier method was used to calculate the overall survival (OS) curves. *P* value of <0.05 (two-sided) was determined to be significant.

## Results

### STOML2 is highly expressed in pancreatic cancer but related to longer survival

**Fig. 1:**
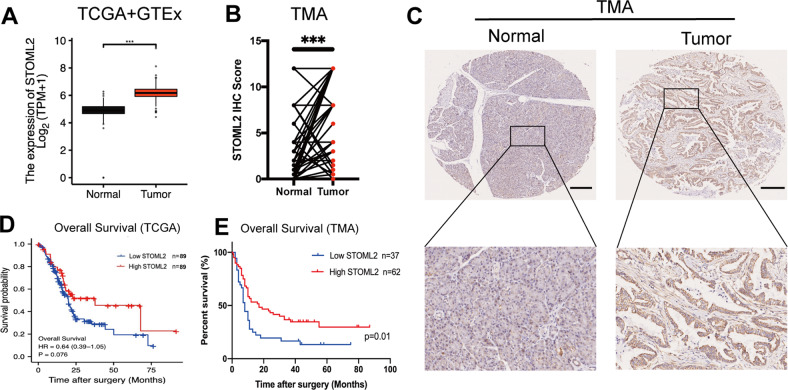
STOML2 was highly expressed in pancreatic cancer tissue but contributed to prolonged survival. **A** Expression levels of STOML2 mRNA in normal pancreas and pancreatic cancer tissue. Data were derived from TCGA and GTEx. **B** IHC scores of STOML2 protein in paired pancreatic cancer tissue and adjacent normal pancreas. **C** Representative IHC results of pancreatic cancer tissue and normal pancreas from tissue microarray (TMA). Scale bar equals 100 μm. **D** Overall survival of low and high STOML2 mRNA groups in TCGA. **E** Overall survival of low and high STOML2 protein groups in the TMA. ***:*P* < 0.001.

To assess the expression of STOML2 in pancreatic cancer and its relationship with overall survival, we analysed STOML2 expression via the TCGA database. The expression of STOML2 in normal pancreatic tissues was also accessed from the GTEx database. Compared to normal pancreatic tissue, STOML2 was highly expressed in pancreatic cancer (Fig. 1A). We further employed clinical samples and tested the STOML2 protein levels in pancreatic cancer tissues by a tissue microarray (TMA). The results supported that STOML2 was highly expressed in pancreatic cancer tissues compared with their paired neighboring normal pancreas tissues (Fig. 1B, C). Next, we explored the correlations between STOML2 expression levels and clinicopathologic parameters among the 99 patients in the TMA. Compared to the male group, STOML2 was highly expressed in the female group (*p* = 0.019; Table 1). Moreover, the association between STOML2 expression and overall survival (OS) time in pancreatic cancer patients was explored via Kaplan‒Meier analysis. The results indicated that the patients in the low STOML2 expression group had significantly shorter OS than those in the high STOML2 expression group in both the TCGA database and TMA (Fig. 1D, E). Hence, STOML2 was remarkably highly expressed in pancreatic cancer, which indicated a good prognosis in pancreatic cancer patients.

**Table 1:** Correlations of STOML2 expression levels with clinical and pathologic parameters.

Variables	*n*	Low STOML2 group *n* = 37	High STOML2 group *n* = 62	*P* value
Gender				0.019*
Male	63	29	34	
Female	36	8	28	
Age				0.469
<60	48	16	32	
≥60	50	21	29	
Location				0.983
Head	59	22	37	
Body/tail	40	15	25	
Differential degree				0.367
Low	32	14	18	
High/moderate	67	23	44	
T stage				0.799
T1/2	61	23	38	
T3	37	13	24	
N stage				0.46
N0	50	20	30	
N1/N2	43	17	26	
TNM stage				0.44
I/IIA	48	19	29	
IIB/III/IV	44	14	30	

### STOML2 inhibits pancreatic cancer cell proliferation and chemoresistance

**Fig. 2:**
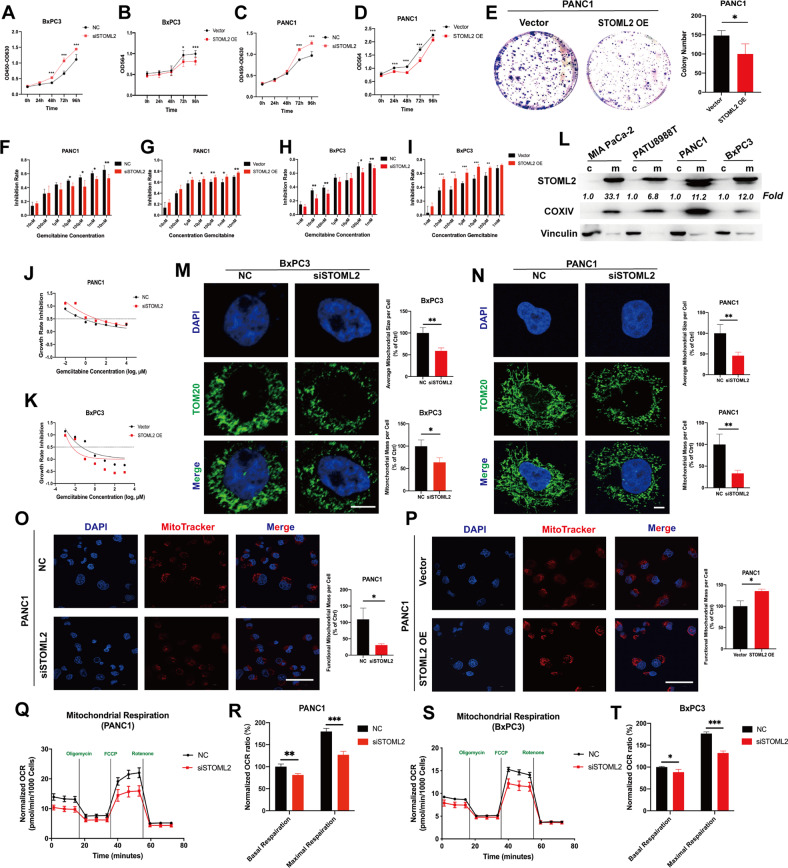
STOML2 repressed proliferation and chemoresistance and was positively related to mitochondrial mass and respiration in pancreatic cancer cells. **A**–**D** The proliferation of BxPC3 and PANC1 cells after downregulating (siSTOML2) or upregulating STOML2 (STOML2 OE). **E** Colony formation of PANC1 cells after STOML2 overexpression. **F**–**I** GEM toxicity in gradient concentrations to BxPC3 and PANC1 cells after downregulating or upregulating STOML2. **J**–**K** The chemoresistance of BxPC3 and PANC1 cells was further investigated by the GR50 assay. **L** Immunoblot showing the protein levels of STOML2 in the cytosol (c) and mitochondria (m) from four pancreatic cancer cell lines, including MIA Paca-2, PATU8988T, PANC1, and BxPC3. **M**–**N** Immunofluorescence staining of mitochondria in BxPC3 and PANC1 cells using anti-TOM20. Scale bars equal 5 μm. **O**–**P** MitoTracker staining showed the functional mitochondrial mass of PANC1 cells after downregulating or upregulating STOML2. Scale bars equal 50 μm. **Q**–**T** Mitochondrial oxidative respiration in BxPC3 and PANC1 cells was measured by Seahorse assay after downregulating STOML2. *P < 0.05; **P < 0.01; ***P < 0.001.

STOML2 was inhibited by small interfering RNA (siSTOML2) and overexpressed by an overexpression plasmid (STOML2 OE) in pancreatic cancer cell lines (PANC1 and BxPC3). The proliferation of pancreatic cancer cells was monitored by SRB assay, CCK8 assay and colony formation assay. These results showed that the cell proliferative ability was significantly decreased after STOML2 overexpression but upregulated after STOML2 inhibition in PANC1 and BxPC3 cells (Fig. 2A–E). Considering that STOML2 expression has a strong relationship with the prognosis of pancreatic cancer patients, we further tested the function of STOML2 in GEM resistance, which is the first-line chemotherapy against pancreatic cancer [21]. In PANC1 and BxPC3 cells, STOML2 inhibition significantly reduced the inhibition rate of GEM treatment at multiple concentrations, while STOML2 overexpression upregulated the inhibition rate under GEM treatment (Fig. 2F–I). To further confirm the role of STOML2 in chemoresistance, a GR50 assay was employed to eliminate potential confounders such as the proliferation rate [19]. According to the formula mentioned in the Methods section, GR50 is the GEM concentration at which growth rate inhibition = 0.5. STOML2 inhibition improved GR50 in PANC1 cells (Fig. 2J), thus increasing chemoresistance. Similarly, STOML2 overexpression decreased GR50 in BxPC3 cells (Fig. 2K). Therefore, STOML2 could inhibit the proliferation and chemoresistance of pancreatic cancer cells.

### STOML2 is primarily located in mitochondria and positively associated with mitochondrial mass and respiration in pancreatic cancer cells

According to previous reports, STOML2 is a membrane‐anchored protein located on the inner membrane of mitochondria [22]. To further verify the localization of STOML2 in pancreatic cancer cells, cytoplasmic proteins (c) and mitochondrial proteins (m) were extracted from four pancreatic cancer cell lines (MIA PaCa-2, PATU8988T, PANC1, and BxPC3). Vinculin and COXIV were chosen as the reference standards for the expression and purity of cytoplasmic and mitochondrial proteins, respectively [23, 24]. The results suggested that STOML2 was primarily located in the mitochondria of pancreatic cancer cells (Fig. 2L). We next investigated the function of STOML2 in mitochondria. Translocase of outer mitochondrial membrane (TOM20) is a specific mitochondrial protein that is often utilized to show mitochondrial content [25, 26]. We employed immunofluorescence and TOM20 to observe the morphological alteration of mitochondria. According to the results, STOML2 downregulation significantly reduced the average mitochondrial size and total mitochondrial mass in both BxPC3 and PANC1 cells (Fig. 2M, N). Thus, STOML2 inhibition might promote mitochondrial fragmentation and mitophagy. Additionally, a lysosomal inhibitor (chloroquine, CQ) was used to further explore the role of mitophagy. The results showed that STOML2 downregulation could still obviously increase mitochondrial fragmentation but failed to reduce the mitochondrial mass in both BxPC3 and PANC1 cells under CQ treatment (Fig. S1A, B), indicating the key role of mitophagy in the mitochondrial content alteration induced by STOML2 downregulation. MitoTracker was used to label mitochondria based on the mitochondrial membrane potential [27]. Therefore, we further analysed the role of STOML2 in functional mitochondrial content via MitoTracker. The results showed that STOML2 inhibition decreased functional mitochondrial mass, while STOML2 overexpression increased functional mitochondrial mass in PANC1 cells (Fig. 2O, P), which further indicated that STOML2 downregulation restricted mitochondrial content. In addition to morphological changes, mitochondrial respiration ability was also investigated. We measured the OCR following STOML2 knockdown. The basal OCR data were acquired before oligomycin treatment (1.5 μM), and the maximal OCR data were acquired after FCCP treatment (2 μM). The results suggested that STOML2 downregulation could significantly decrease basal and maximal mitochondrial respiration in both PANC1 and BxPC3 cells (Fig. 2Q–T). Therefore, STOML2 might repress mitophagy and increase functional mitochondrial mass, potentially decreasing GEM resistance in pancreatic cancer cells [28].

### STOML2 restricts full-length PINK1 and mitophagy in pancreatic cancer cells

**Fig. 3:**
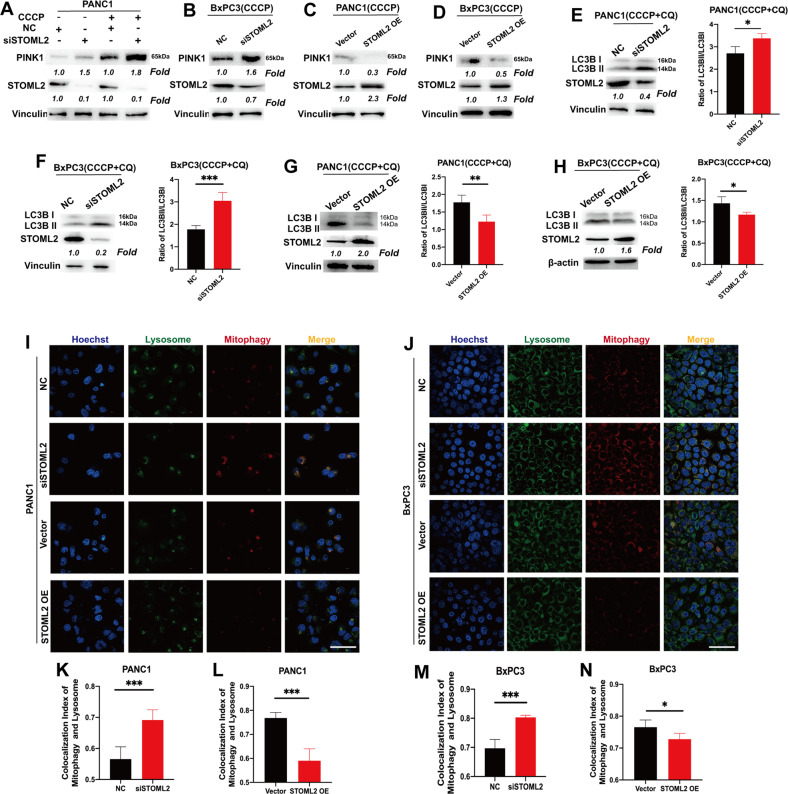
STOML2 repressed PINK1-induced mitophagy in pancreatic cancer cells. **A**–**D** Immunoblot showing the protein levels of PINK1, STOML2 and Vinculin in PANC1 and BxPC3 cells after downregulating or upregulating STOML2, which were treated with CCCP (10 μM) or vehicle in cell medium for 24 h. **E**–**H** Immunoblot showing the protein levels of LC3BI, LC3BII, STOML2, and Vinculin in PANC1 and BxPC3 cells after downregulating or upregulating STOML2, which were treated with CQ (10 μM) and CCCP (10 μM) in cell medium for 24 h. The ratios of LC3BII/LC3BI were calculated by the OD value of the corresponding blot band determined by ImageJ. **I**–**J** Fluorescent staining showed the colocalization between lysosomes and mitophagy (mitochondria) in PANC1 and BxPC3 cells after downregulating or upregulating STOML2. The quantitative results were calculated by CellProfiler V4.2.1. Scale bar equals 50 μm. *P < 0.05; **P < 0.01; ***P < 0.001.

We further assessed the relationship between STOML2 expression and PINK1-mediated mitophagy. There are many isoforms of PINK1 with different lengths, but only full-length PINK1 (approximately 65 kDa) can functionally recruit Parkin and induce mitophagy [29]. Because the expression of full-length PINK1 was low in the general state (Fig. 3A), we employed carbonyl cyanide m-chlorophenylhydrazone (CCCP) (10 μM) to elevate PINK1 levels, making it suitable to observe the effects of STOML2 on PINK1 in pancreatic cancer cells [30]. After downregulating STOML2 in PANC1 and BxPC3 cells, full-length PINK1 was upregulated (Fig. 3A, B), while overexpressing STOML2 inhibited the full-length PINK1 levels in PANC1 and BxPC3 cells (Fig. 3C, D). Given that mitophagy is a part of autophagy, we further investigated autophagic flux, which could partially reflect mitophagy levels [31]. CQ (10 μM) treatment was used to block autophagic flux, as reflected in the ratio of LC3BII/LC3BI [32]. Downregulating STOML2 in both PANC1 and BxPC3 cells significantly increased the LC3BII/LC3BI ratio (Fig. 3E, F), while upregulating STOML2 decreased the LC3BII/LC3BI ratio (Fig. 3G, H). Therefore, these results suggested that STOML2 repressed full-length PINK1 and overall autophagy levels in pancreatic cancer cells, which indicated the key role of STOML2 in mitophagy.

To further confirm the function of STOML2 in mitophagy, we utilized lysosome dye and mitophagy dye to detect mitophagy levels in PANC1 and BxPC3 cells [33]. After inhibiting STOML2 in PANC1 and BxPC3 cells, the intensity of mitophagy and the area of colocalization between lysosomes and mitophagy were enhanced. Consistently, overexpressing STOML2 decreased the intensity of mitophagy and the area of colocalization between lysosomes and mitophagy (Fig. 3I–N). Therefore, STOML2 could effectively restrict mitophagy in pancreatic cancer cells, which might mediate its role in reducing chemoresistance and prolonging OS in pancreatic cancer patients.

### STOML2 alleviates GEM-induced PINK1 elevation and mitophagy-mediated chemoresistance by stabilizing PARL

A previous study proved that GEM treatment could improve PINK1-induced mitophagy [34]. To ensure the relationship between GEM and mitophagy in pancreatic cancer cells, GEM was added to the culture medium of PANC1 (10 μM) and BxPC3 (1 μM) cells. The expression levels of PINK1 were investigated after 48 h. GEM treatment elevated the levels of full-length PINK1 (Fig. S2A, B). The results suggested that GEM could elevate the basic autophagy level in pancreatic cancer cells (Fig. S2C, D). We next utilized lysosome dye and mitophagy dye to further detect mitophagy levels. Consistently, after GEM administration, mitophagy staining was enhanced, and the colocalization area between lysosomes and mitophagy was also significantly increased (Fig. S2E, F). Hence, GEM could promote PINK1-induced mitophagy in pancreatic cancer cells.

**Fig. 4:**
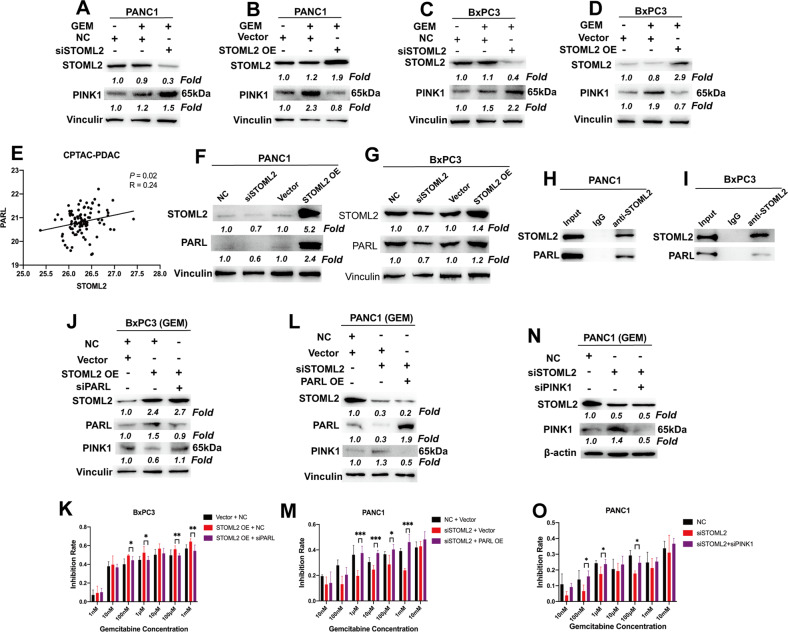
STOML2 repressed PINK1-induced mitophagy and chemoresistance by stabilizing PARL. **A**–**D** Immunoblot showing the protein levels of STOML2, PINK1 and Vinculin in PANC1 and BxPC3 cells after downregulating or upregulating STOML2, which were treated with GEM or vehicle in cell medium for 24 h. **E** The correlation between PARL and STOML2 protein levels in pancreatic cancer. Pearson correlation coefficients were employed to quantify the correlation. **F**, **G** Immunoblot showing the protein levels of STOML2, PARL and Vinculin in PANC1 and BxPC3 cells after downregulating or upregulating STOML2. **H**, **I** Coimmunoprecipitation showed that STOML2 and PARL were pulled down by the STOML2 antibody in PANC1 and BxPC3 cells. **J** Immunoblot showing the protein levels of STOML2, PARL, PINK1 and Vinculin in BxPC3 cells after upregulating STOML2 and downregulating PARL (STOML2 OE + siPARL) compared to the control groups, which were treated with GEM (1 μM) in cell medium for 24 h. **K** GEM toxicity to BxPC3 cells after overexpressing STOML2 and downregulating PARL compared to the control groups. **L** Immunoblot showing the protein levels of STOML2, PARL, PINK1 and Vinculin in PANC1 cells after downregulating STOML2 and upregulating PARL (siSTOML2 + PARL OE) compared to the control groups, which were treated with GEM (10 μM) in cell medium for 24 h. **M** GEM toxicity to PANC1 cells after downregulating STOML2 and upregulating PARL compared to the control groups. **N** Immunoblot showing the protein levels of STOML2, PINK1 and Vinculin in PANC1 cells after downregulating STOML2 and PINK1 (siSTOML2 + siPINK1) compared to the control groups, which were treated with GEM (10 μM) in cell medium for 24 h. **O** GEM toxicity to PANC1 cells after downregulating STOML2 and PINK1 compared to the control groups. *P < 0.05; **P < 0.01; ***P < 0.001.

To explore whether STOML2 repressed GEM-induced PINK1 elevation and chemoresistance, we investigated PINK1 under GEM treatment after downregulating/upregulating STOML2. Compared to the control groups, downregulating STOML2 could further increase PINK1, while upregulating STOML2 reversed the elevation of PINK1 caused by GEM (10 μM for PANC1 and 1 μM for BxPC3) (Fig. 4A–D). Previous reports suggested that STOML2 could directly stabilize PARL, a kind of protease that could degrade PINK1 [22]. We thus tested the association between STOML2 and PARL. Based on the proteomic data of 140 pancreatic cancer cases [15], there was a significant positive association between STOML2 and PARL at the protein level (Fig. 4E, *P* = 0.02, R = 0.24). After altering the expression levels of STOML2, the expression of PARL was also changed along with that of STOML2 in PANC1 and BxPC3 cells (Fig. 4F, G). Next, we investigated the direct interaction between STOML2 and PARL by Co-IP. In both PANC1 and BxPC3 cells, the STOML2 antibody could also pull down PARL, which suggested that STOML2 could directly bind and stabilize PARL in pancreatic cancer cells (Fig. 4H, I). To verify the STOML2/PARL/PINK1 pathway and its role in chemoresistance, rescue experiments were performed. After STOML2 overexpression, inhibiting PARL reversed the downregulation of PINK1 (Fig. 4J). The chemosensitivity phenotype driven by STOML2 overexpression was also reversed by PARL downregulation (Fig. 4K). Similarly, after STOML2 downregulation, overexpressing PARL significantly reduced PINK1 expression and chemoresistance (Fig. 4L, M). Consistently, inhibiting PINK1 reversed the chemoresistance phenotype under STOML2 downregulation (Fig. 4N, O). Hence, STOML2 repressed mitophagy-mediated chemoresistance through the STOML2/PARL/PINK1 pathway.

### STOML2 decreases chemoresistance by promoting apoptosis of pancreatic cancer cells

**Fig. 5:**
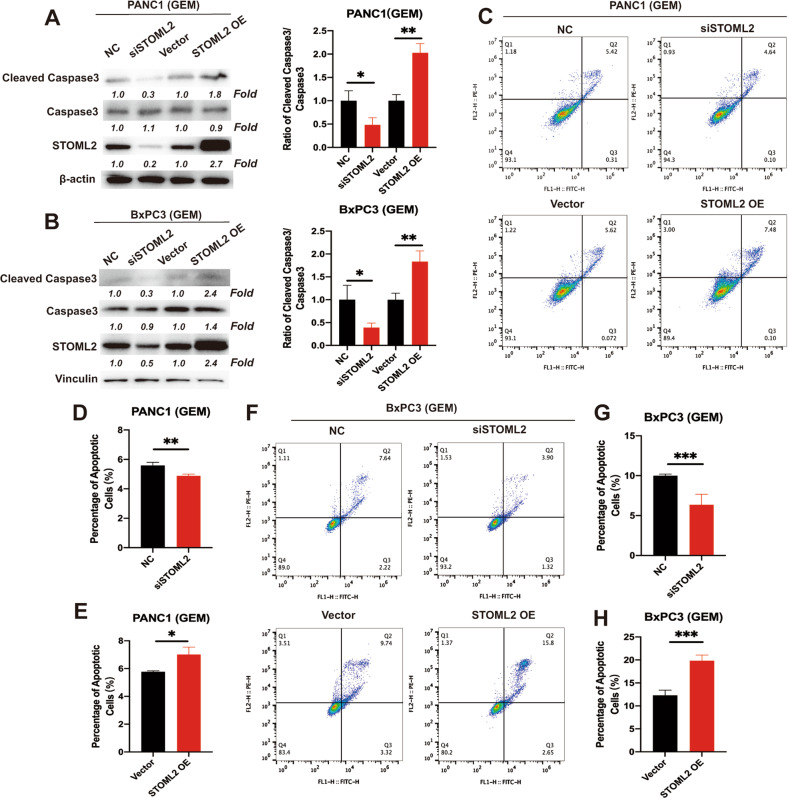
STOML2 promoted GEM-induced apoptosis in pancreatic cancer cells. **A**, **B** Immunoblot showing the protein levels of cleaved caspase 3, caspase 3, STOML2, and Vinculin in PANC1 and BxPC3 cells after downregulating or upregulating STOML2, which were treated with GEM in cell medium for 24 h. **C** Dot plot from the flow cytometry staining to gate the apoptotic PANC1 cells after downregulating or upregulating STOML2, which were treated with GEM (10 μM) in cell medium for 24 h. **D**, **E** The corresponding quantitative data of early and late apoptotic cells in PANC1. **F** Dot plot from the flow cytometry staining to gate the apoptotic BxPC3 cells after downregulating or upregulating STOML2, which were treated with GEM (1 μM) for 24 h. **G**–**H** The corresponding quantitative data of early and late apoptotic BxPC3 cells. *P < 0.05; **P < 0.01; ***P < 0.001.

To further explore the mechanism by which STOML2 inhibited chemoresistance, we examined the apoptosis levels of PANC1 and BxPC3 cells induced by GEM treatment. Compared with the control groups, STOML2 downregulation reduced the proportion of cleaved caspase3 to full-length caspase3, while STOML2 overexpression increased the proportion of cleaved caspase3 to full-length caspase3 (Fig. 5A, B). Therefore, STOML2 promoted GEM-induced apoptosis at the protein level. We further employed flow cytometry and GEM treatment (10 μM for PANC1 and 1 μM for BxPC3) to confirm the role of STOML2 in cell apoptosis. Consistently, after downregulating STOML2, the number of apoptotic cells (including early and late apoptotic cells) was significantly decreased, while overexpressing STOML2 obviously increased the number of apoptotic cells (Fig. 5C–H). These results suggested that STOML2 could promote GEM-induced apoptosis in pancreatic cancer cells.

Extensive cell mitophagy could result in excessive mitochondria loss and cell apoptosis [35]; however, PINK1-induced mitophagy could elevate the mitochondrial biogenesis pathway [36]. Therefore, we investigated the expression levels of key proteins in mitochondrial biogenesis, including NRF1, TFAM, and PGC1α. Downregulating STOML2 in PANC1 and BxPC3 cells upregulated the expression of PGC1α, NRF1, and TFAM at both the RNA and protein levels (Fig. S3A–D). Consistently, upregulating STOML2 in PANC1 and BxPC3 cells downregulated the expression levels of NRF1, TFAM, and PGC1α (Fig. S3E, F). Hence, STOML2 downregulation could also enhance dynamic mitochondrial turnover, which could further prevent excessive mitophagy and maintain cancer cell survival. Oxidative phosphorylation in functional mitochondria is the main source of intracellular ROS [37]. In particular, recent evidence has shown that accumulated damaged mitochondria with compromised mitophagy can generate amounts of ROS [38, 39]. High levels of intracellular ROS can induce apoptosis [40]. Hence, we measured ROS levels after downregulating or overexpressing STOML2 in PANC1 and BxPC3 cells. Downregulating STOML2 significantly reduced ROS levels, and upregulating STOML2 increased ROS levels (Fig. S4A–F). These results suggested that pancreatic cancer cells with high STOML2 expression levels preferentially have compromised mitophagy, accumulated functional mitochondria and higher intracellular ROS levels, which might contribute to GEM-induced apoptosis. To further explore the relationship between STOML2-related ROS elevation and apoptosis, we treated cells with an antioxidant (Trolox, 100 μM) after STOML2 overexpression [41]. The results showed that antioxidant could obviously eliminate ROS levels (Fig. S4G) and significantly reverse STOML2-induced apoptosis in both PANC1 and BxPC3 cells under GEM treatment (100 μM for PANC1 and 10 μM for BxPC3) (Fig. S4H). Interestingly, the apoptosis in STOML2-overexpressing pancreatic cancer cells with antioxidant treatment was still higher than that in the blank control groups (Fig. S4H), which indicated that there might exist other ways to induce mitophagy-related apoptosis. For instance, cytochrome c released from redundant mitochondria to the cytosol could directly trigger the mitochondrial-mediated apoptosis pathway in cells with compromised mitophagy [42, 43].

### STOML2 decreases the chemoresistance of pancreatic cancer in vivo

**Fig. 6:**
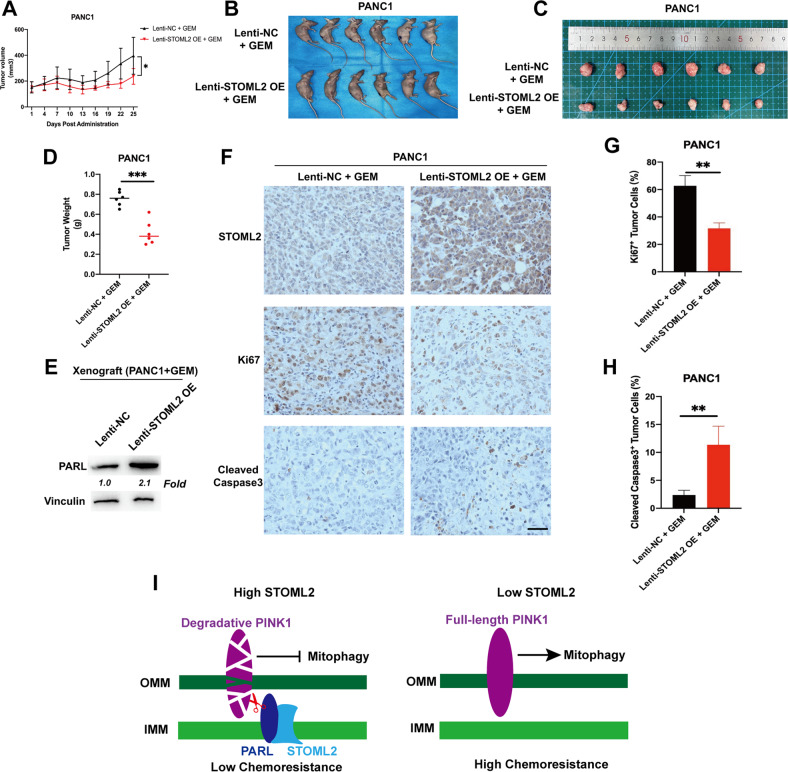
STOML2 repressed GEM chemoresistance in vivo. **A** Under GEM treatment, subcutaneous xenograft tumors with STOML2 overexpression had a lower growth rate than the control group. **B** Representative figures of tumors in nude mice (*N* = 7/group). **C** Subcutaneous xenograft tumors derived from PANC1-lenti-NC cells or PANC1-lenti-STOML2 OE cells. **D** Tumor weights of the subcutaneous xenograft tumors. **E** Immunoblot showing the protein levels of PARL and vinculin after STOML2 overexpression in subcutaneous xenograft tumors. **F** IHC confirmed STOML2 overexpression in PANC1-lenti-STOML2 OE cells compared to control cells. **F**–**H** Cell proliferation and cell death in vivo were also analysed by IHC using anti-Ki67 and cleaved caspase 3, respectively. Scale bar represents 50 μm **G** Graphical diagram of STOML2-inhibited mitophagy-induced chemoresistance in pancreatic cancer cells through the STOML2/PARL/PINK1 axis. OMM: outer mitochondrial membrane; IMM: inner mitochondrial membrane. *P < 0.05; **P < 0.01; ***P < 0.001.

To validate the function of STOML2 in decreasing chemoresistance in vivo, we employed nude mice and stable STOML2-overexpressing PANC1 cells to construct subcutaneous xenografts. After all of the individual volume subcutaneous xenografts reached 80 mm^3^, the qualified mice were injected with GEM, and the tumor volumes were measured every three days (Fig. 6A). Four weeks after GEM administration, the involved mice were sacrificed (Fig. 6B). The tumor volume and tumor weight were directly compared between the two groups (Fig. 6C, D). These results showed that STOML2 obviously decreased tumor growth under GEM treatment in vivo. Thus, STOML2 could effectively reduce the chemoresistance of pancreatic cancer in vivo. STOML2 expression and downstream PARL were further verified in vivo (Fig. 6E, F), indicating stable STOML2 overexpression and the STOML2/PARL pathway in vivo. Additionally, the proliferation and cell death in xenografts were also analysed by IHC. Ki67 and cleaved caspase3 are markers for proliferation and cell death, respectively [44]. The results suggested that STOML2 overexpression could significantly repress cell proliferation and promote cell death under GEM treatment in vivo (Fig. 6F, H). In conclusion, STOML2 could stabilize PARL and promote PINK1 degradation, which represses mitophagy and ultimately reduces chemoresistance in pancreatic cancer (Fig. 6I).

## Discussion

Pancreatic cancer remains one of the most lethal diseases at present. Despite decades of research, chemotherapy resistance remains a thorny issue limiting the survival time of pancreatic cancer patients. There are many mechanisms by which pancreatic cancer cells induce chemoresistance [45]. Recently, accumulated studies have suggested that the mass and quality of mitochondria are vital for pancreatic cancer cell survival, which also contributes to the dynamic adaptation to adverse environmental conditions, such as starvation, hypoxia and chemotherapy treatment [4]. Mitochondria are not only major energy providers but also the site of the tricarboxylic acid cycle and amino acid and lipid metabolism [2]. As an organelle, the status of individual mitochondria is highly different. Mitophagy is an essential process for the removal of redundant mitochondria and plays an important role in many physiological and pathological processes. However, mitophagy plays a dual and complex role in carcinogenesis and cancer progression [39, 46]. In this study, we reported that STOML2 was highly expressed in pancreatic tumor tissue but related to longer survival, which might support the idea that mitophagy could repress tumorigenesis but promote cancer progression [47, 48]. The underlying mechanism was also explored in this study. STOML2 could directly stabilize PARL, which could degrade PINK1 and inhibit its role in inducing mitophagy. Simultaneously, STOML2 inhibited autophagic flux in pancreatic cancer, which might contribute to chemoresistance in pancreatic cancer [49]. Consequently, ROS levels and apoptosis were significantly increased in pancreatic cancer cells, ultimately leading to reduced chemoresistance and prolonged survival.

For chemoresistance, previous studies suggested that impaired mitophagy restricted metabolic plasticity, which made pancreatic cancer cells susceptible to metformin treatment [50]. However, another related study showed that mitophagy could inhibit malignant behaviors, including chemoresistance, in pancreatic cancer cells [51]. Therefore, there remain many controversies between mitophagy and chemoresistance in pancreatic cancer, which deserves further study [52]. Mitochondrial respiration is the main source of intracellular ROS [53, 54]. After excessive production, excess ROS directly changes the mitochondrial membrane structure, contributing to the leakage of ROS from mitochondria. As a result, relatively high levels of ROS damage nuclear DNA and even cause strand breaks, ultimately promoting genomic instability [55]. During cancer progression, ROS is also a double-edged sword. Some reports have shown that certain levels of ROS can stimulate cancer proliferation and metastasis, which is partially mediated by inactivating tumor suppressor phosphatases. However, high levels of ROS are cytotoxic, resulting in apoptosis [56]. Therefore, STOML2 might extensively inhibit the clearance of redundant mitochondria, which leads to increased intracellular ROS levels and subsequent cell cycle arrest and apoptosis [57, 58]. In addition to ROS, other mechanisms that mediate mitophagy and apoptosis also deserve further study [42, 43].

However, too much mitophagy results in mitochondrial deficiency, which cannot supply enough energy and metabolites for cell survival. Therefore, in addition to mitophagy, mitochondrial biogenesis is also necessary for maintaining healthy mitochondrial mass and respiratory capacity, assisting cancer cell survival and metastasis [59]. Multiple regulators also participate in mitochondrial biogenesis. A previous study suggested that mitophagy could promote the translocation of transcription factor EB (TFEB) to the nucleus, which induced the transcription of PGC1α mRNA [60]. PGC1α is identified as a key factor [61] to activate nuclear respiratory factor (NRF1) and then increase the expression of nuclear mitochondrial transcription factor gene (TFAM) [62]. In this study, we found that mitophagy driven by low STOML2 could promote the expression levels of PGC1α, NRF1, and TFAM, which indicated that low STOML2-promoted mitophagy could also induce mitochondrial biogenesis. Young mitochondria could provide sufficient energy but produce lower levels of ROS, which might further promote cell survival and chemoresistance.

Other related studies have also reported the role of STOML2 in cancer. In hepatocellular cancer, STOML2 could directly bind with PINK1 and promote its subsequent mitophagy [10], which is different from our present study in pancreatic cancer. The difference in gene mutation and expression between hepatocellular cancer cells and pancreatic cancer cells might contribute to the distinct role of STOML2 in mitophagy. Another report showed that STOML2 could upregulate the hexosamine biosynthetic pathway and promote pancreatic cancer metastasis [14]. However, the function of STOML2 in mitophagy and chemoresistance was not investigated in that study. Proteins might contribute to different phenotypes via distinct molecular pathways. This study also has some shortcomings. First, we only analysed STOML2-overexpressing pancreatic cancer cells in vivo, which lacked data from the STOML2 downregulation group. Second, the detailed peptide sequence of STOML2 that mediates the direct contact between PARL and PINK1 should be addressed, which might provide more evidence for the STOML2/PARL pathway. In conclusion, our study found that STOML2 could repress PINK1-induced mitophagy by directly stabilizing PARL, thus inhibiting pancreatic cancer chemoresistance. Considering the development of targeted drug delivery, it is possible to construct engineered exosomes with exogenous nucleic acids encoding STOML2 or STOML2 protein that could deliver them to pancreatic cancer cells in patients [63]. Therefore, overexpression of STOML2 might be a novel anticancer strategy for pancreatic cancer treatment.

## Supplementary information

original data files-WB

Supplemental Figure 1

Supplemental Figure 2

Supplemental Figure 3

Supplemental Figure 4

Supplemental Table 1

Supplementary Figure legends

Reproducibility checklist
