# Supplementary material for: STOML2 restricts mitophagy and increases chemosensitivity in pancreatic cancer through stabilizing PARL-induced PINK1 degradation
Source: Cell Death Dis. 2023 Mar 11;14(3):191. doi: 10.1038/s41419-023-05711-5 (PMC10008575; doi:10.1038/s41419-023-05711-5)

Figure 2L

COX4

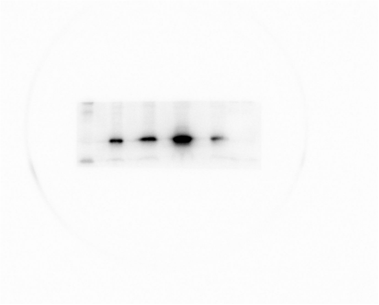

STOML2

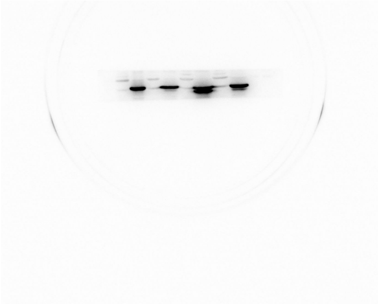

Vinculin

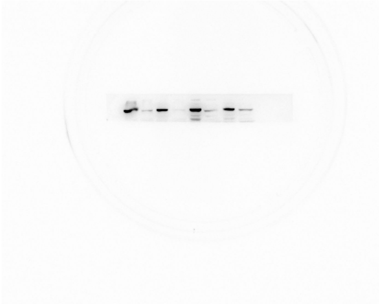

Figure 3A

PINK1

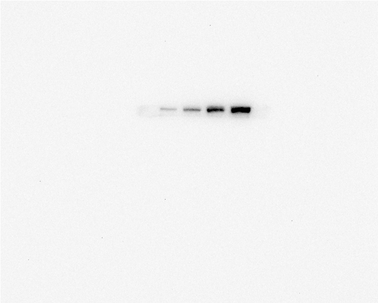

STOML2

Vinculin

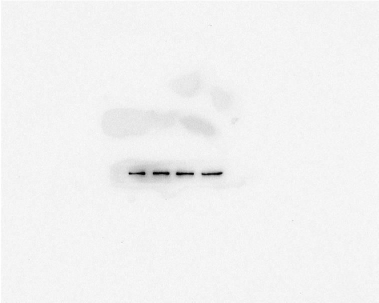

Figure 3B

PINK1

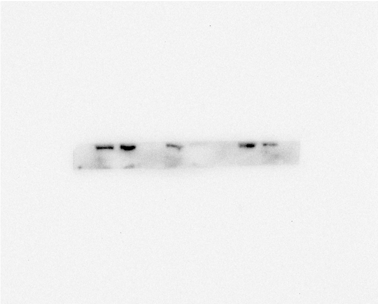

STOML2

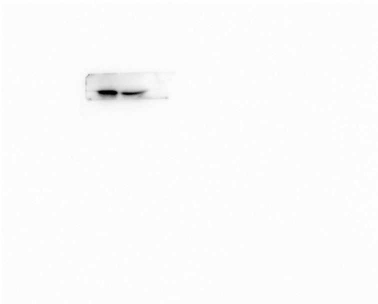

Vinculin

Figure 3C

PINK1

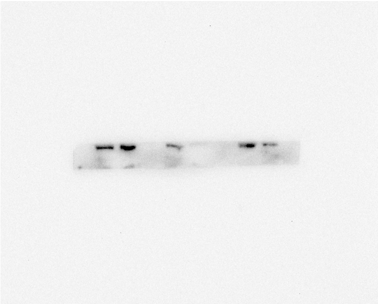

STOML2

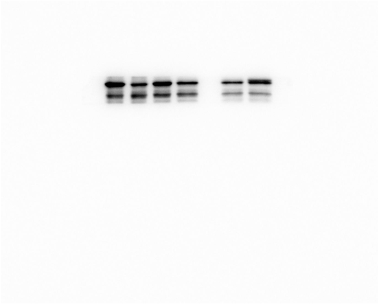

Vinculin

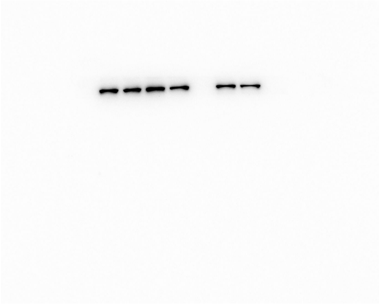

Figure 3D

PINK1

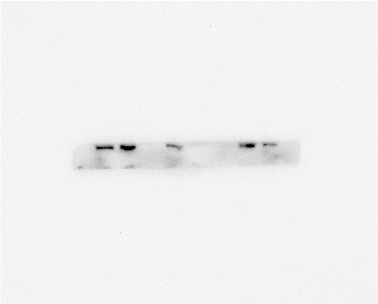

STOML2

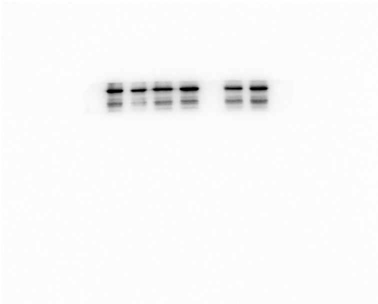

Vinculin

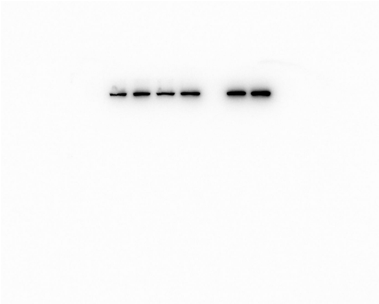

Figure 3E

LC3

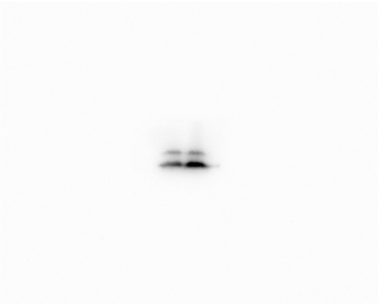

STOML2

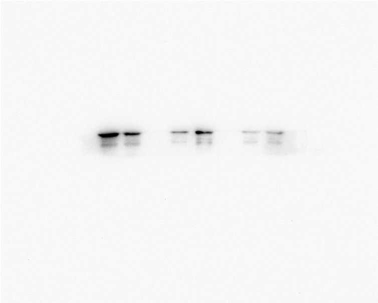

Vinculin

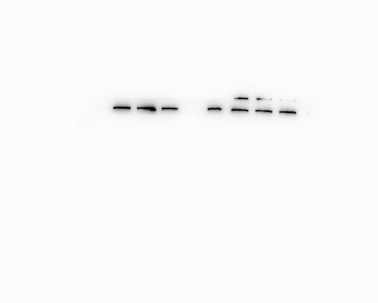

Figure 3F

LC3

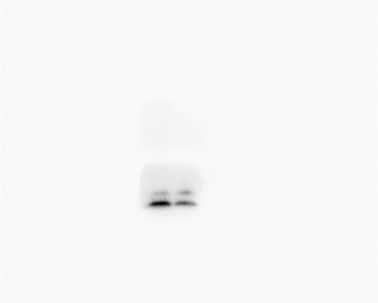

STOML2

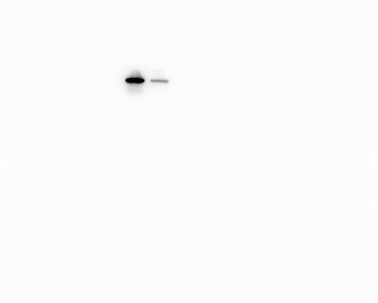

Vinculin

Figure 3G

LC3

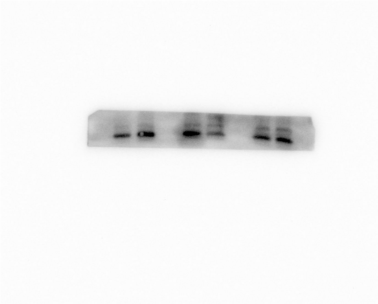

STOML2

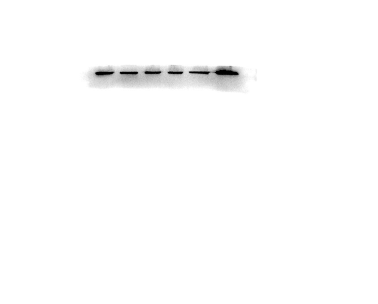

Vinculin

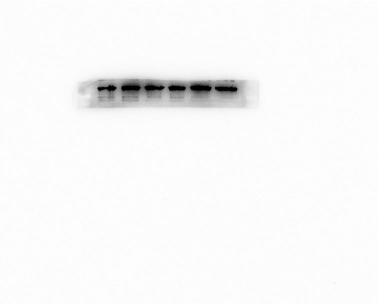

Figure 3H

LC3

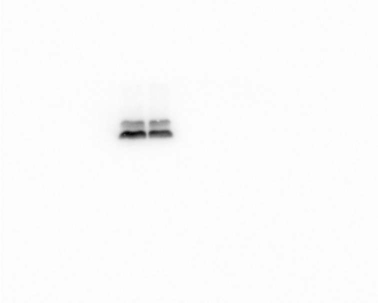

STOML2

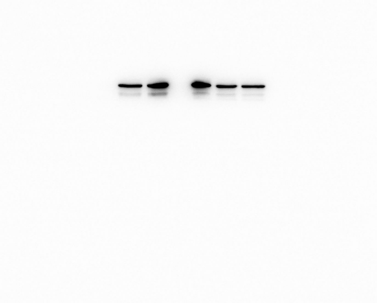

β-actin

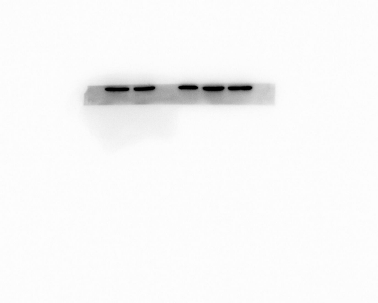

Figure 4A

PINK1

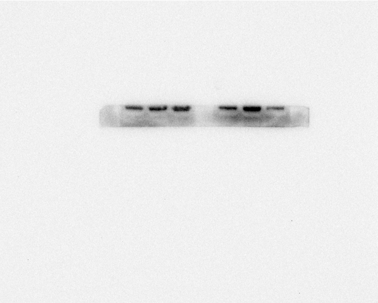

STOML2

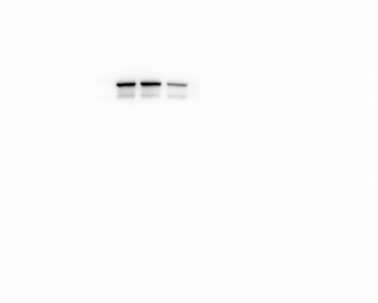

Vinculin

Figure 4B

PINK1

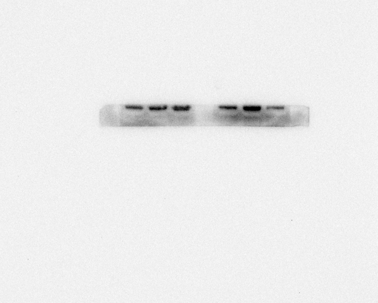

STOML2

Vinculin

Figure 4C

PINK1

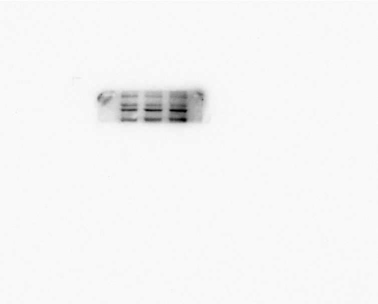

STOML2

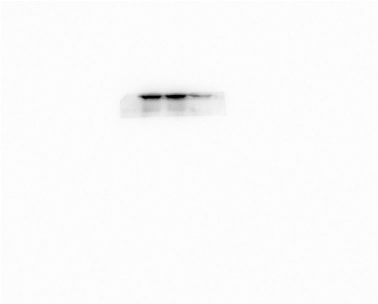

Vinculin

Figure 4D

PINK1

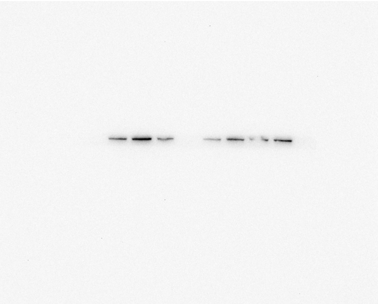

STOML2

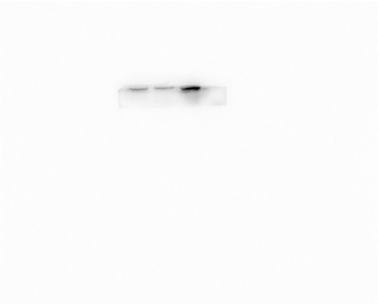

Vinculin

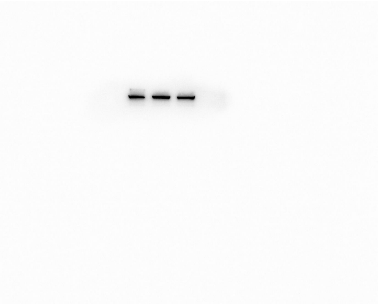

Figure 4F

PARL

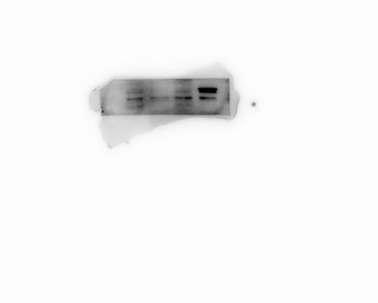

STOML2

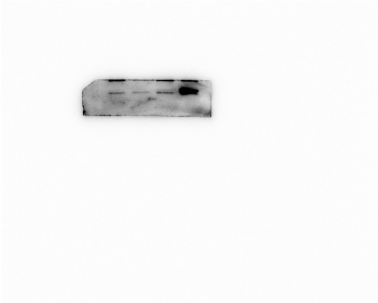

Vinculin

Figure 4G

PARL

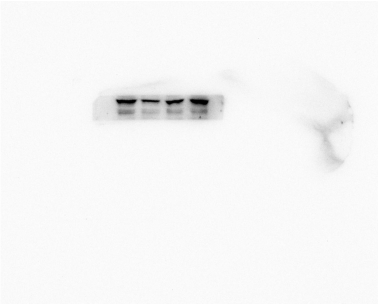

STOML2

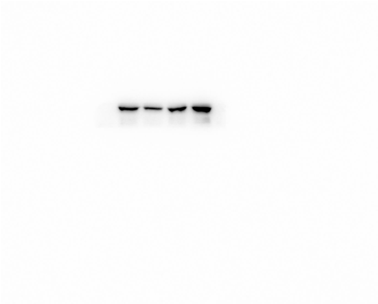

Vinculin

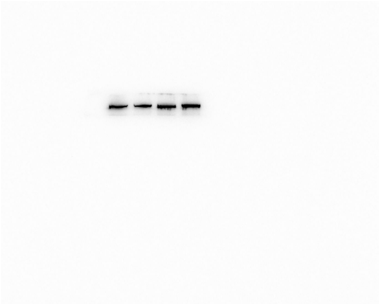

Figure 4H

PARL

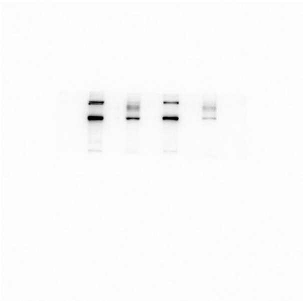

STOML2

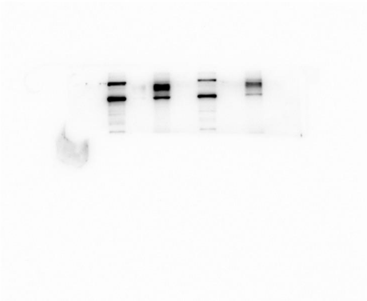

Figure 4I

PARL

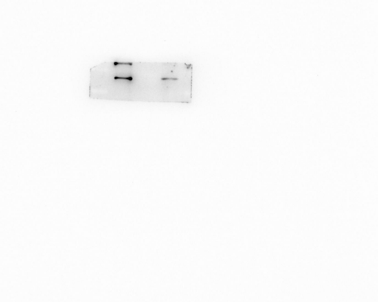

STOML2

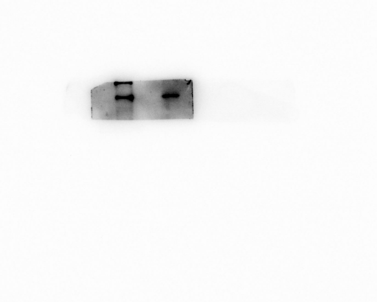

Figure 4J

PARL

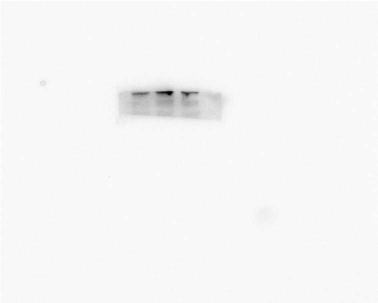

STOML2

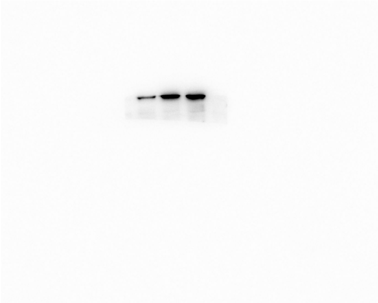

Vinculin

PINK1

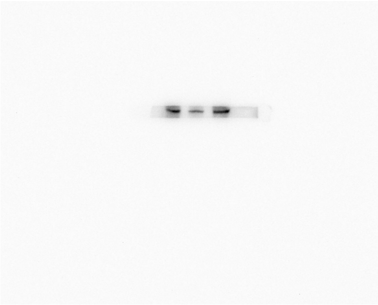

Figure 4L

PINK1

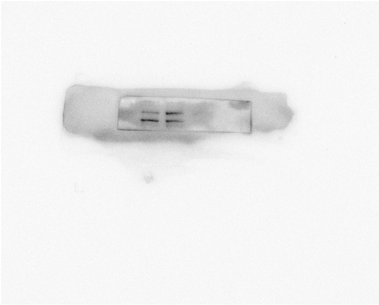

PARL

STOML2

Vinculin

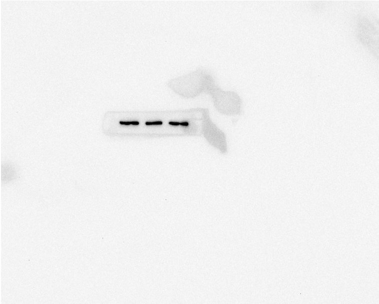

Figure 4N

PINK1

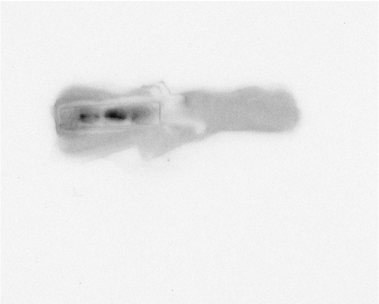

STOML2

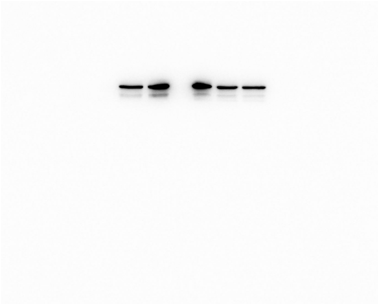

β-actin

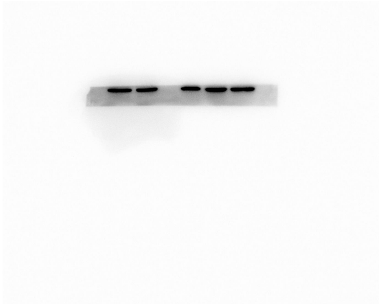

Figure 5A

caspase-3

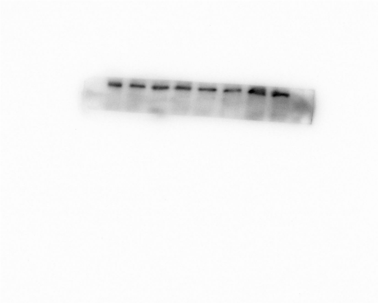

STOML2

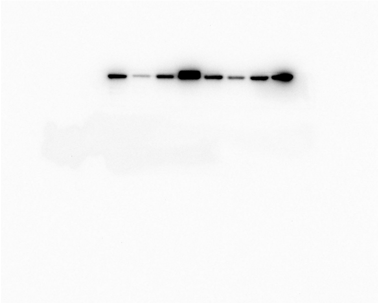

β-actin

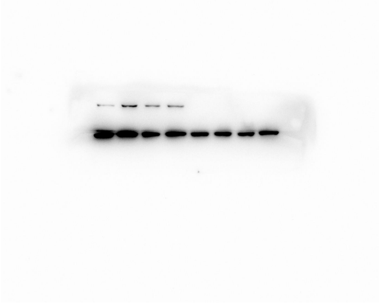

cleaved caspase-3

Figure 5B

cleaved caspase-3

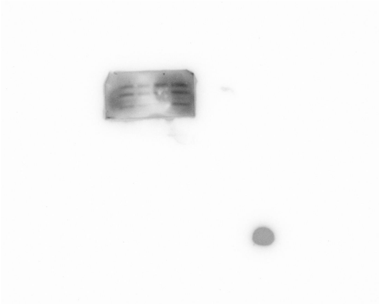

caspase-3

STOML2

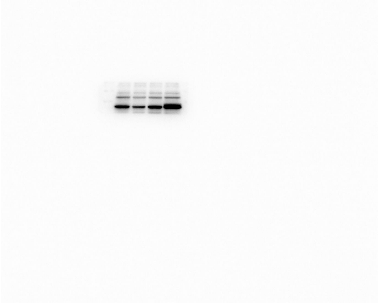

Vinculin

Figure 6E

PARL

Vinculin

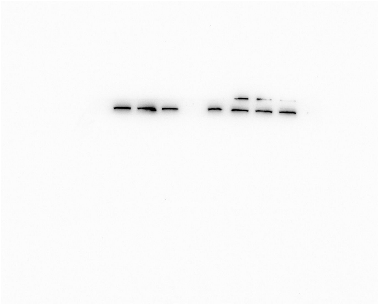

Supplementary Figure 2A

PINK1

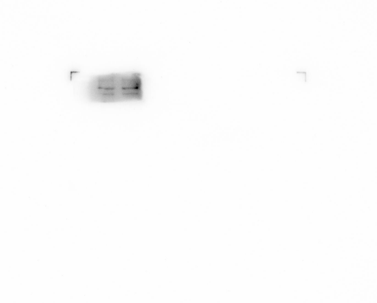

β-actin

Supplementary Figure 2B

PINK1

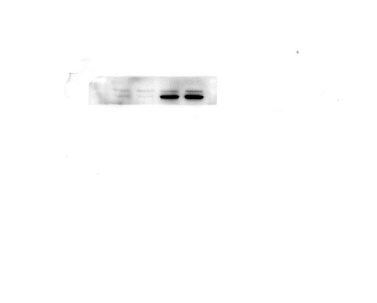

β-actin

Supplementary Figure 2C

LC3B

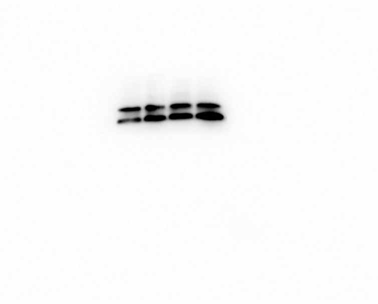

β-actin

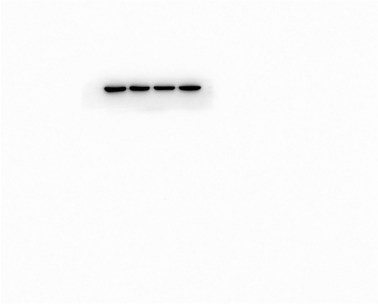

Supplementary Figure 2D

LC3B

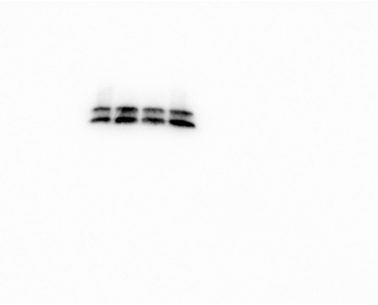

β-actin

Supplementary Figure 3A

NRF1

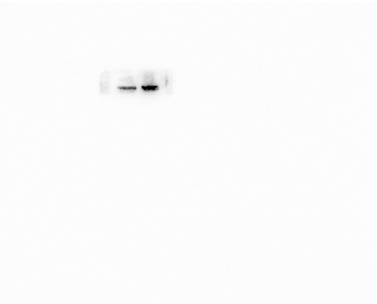

PGC1

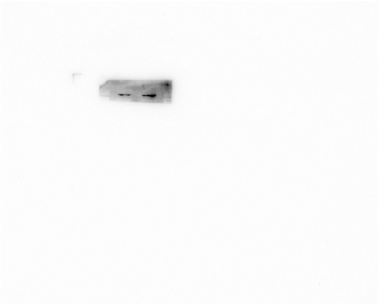

Vinculin

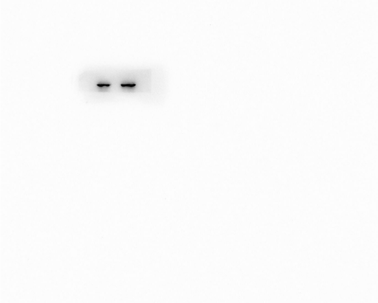

TFAM

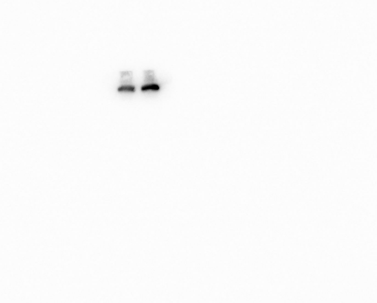

STOML2

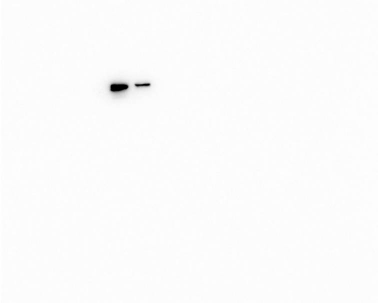

Supplementary Figure 3B

NRF1

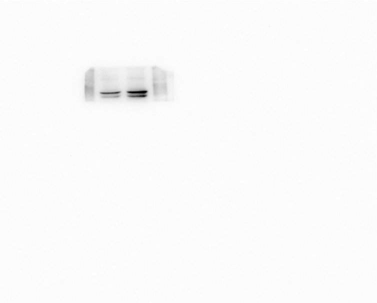

PGC1

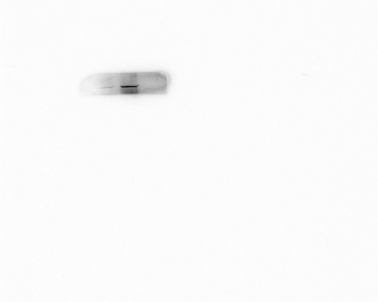

β-actin

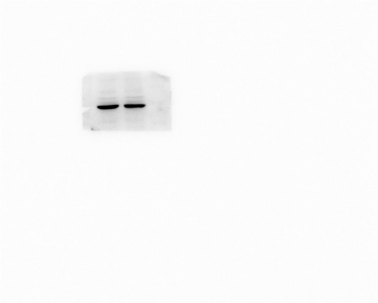

TFAM

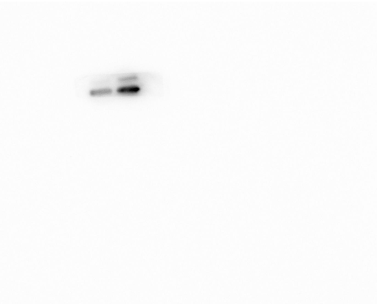

STOML2

Supplementary Figure 3E

NRF1

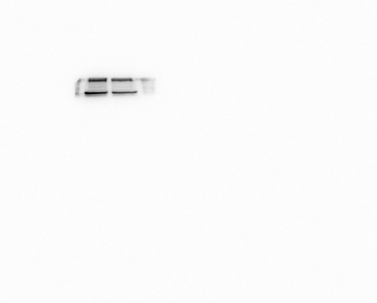

PGC1

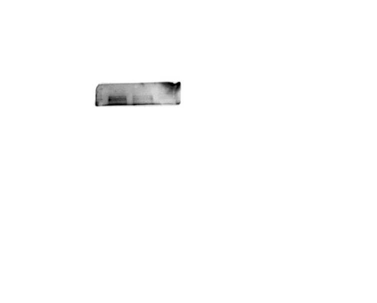

Vinculin

TFAM

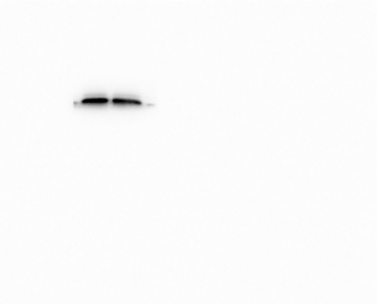

STOML2

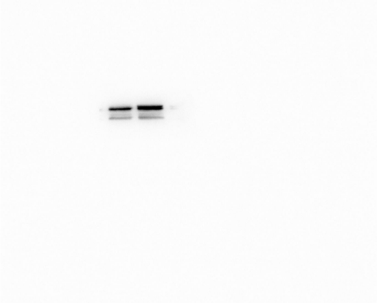

Supplementary Figure 3F

NRF1

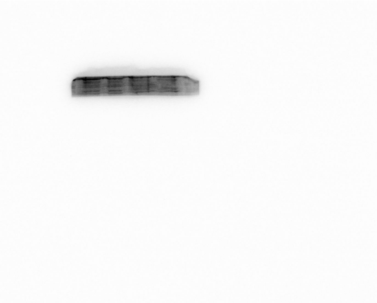

PGC1

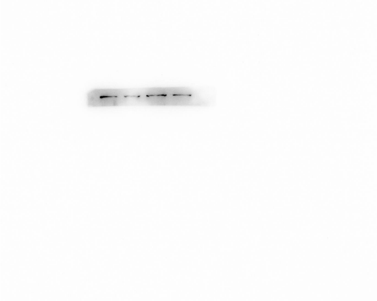

Vinculin

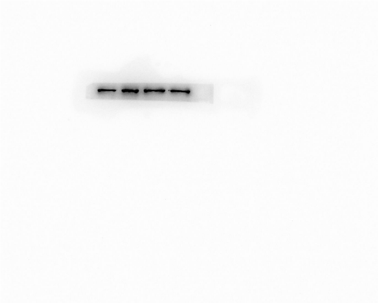

TFAM

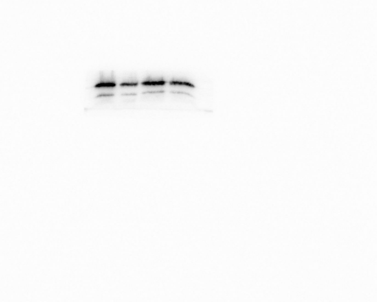

STOML2

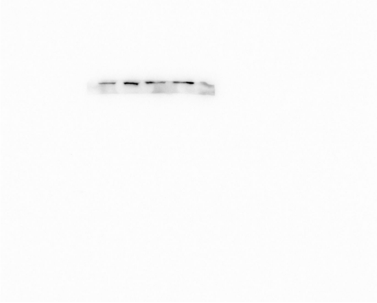

Supplement: Supplementary file 1 — original data files-WB [file 41419_2023_5711_MOESM1_ESM.pdf]
