# Supplementary figures and images for: STOML2 restricts mitophagy and increases chemosensitivity in pancreatic cancer through stabilizing PARL-induced PINK1 degradation
Source: Cell Death Dis. 2023 Mar 11;14(3):191. doi: 10.1038/s41419-023-05711-5 (PMC10008575; doi:10.1038/s41419-023-05711-5)

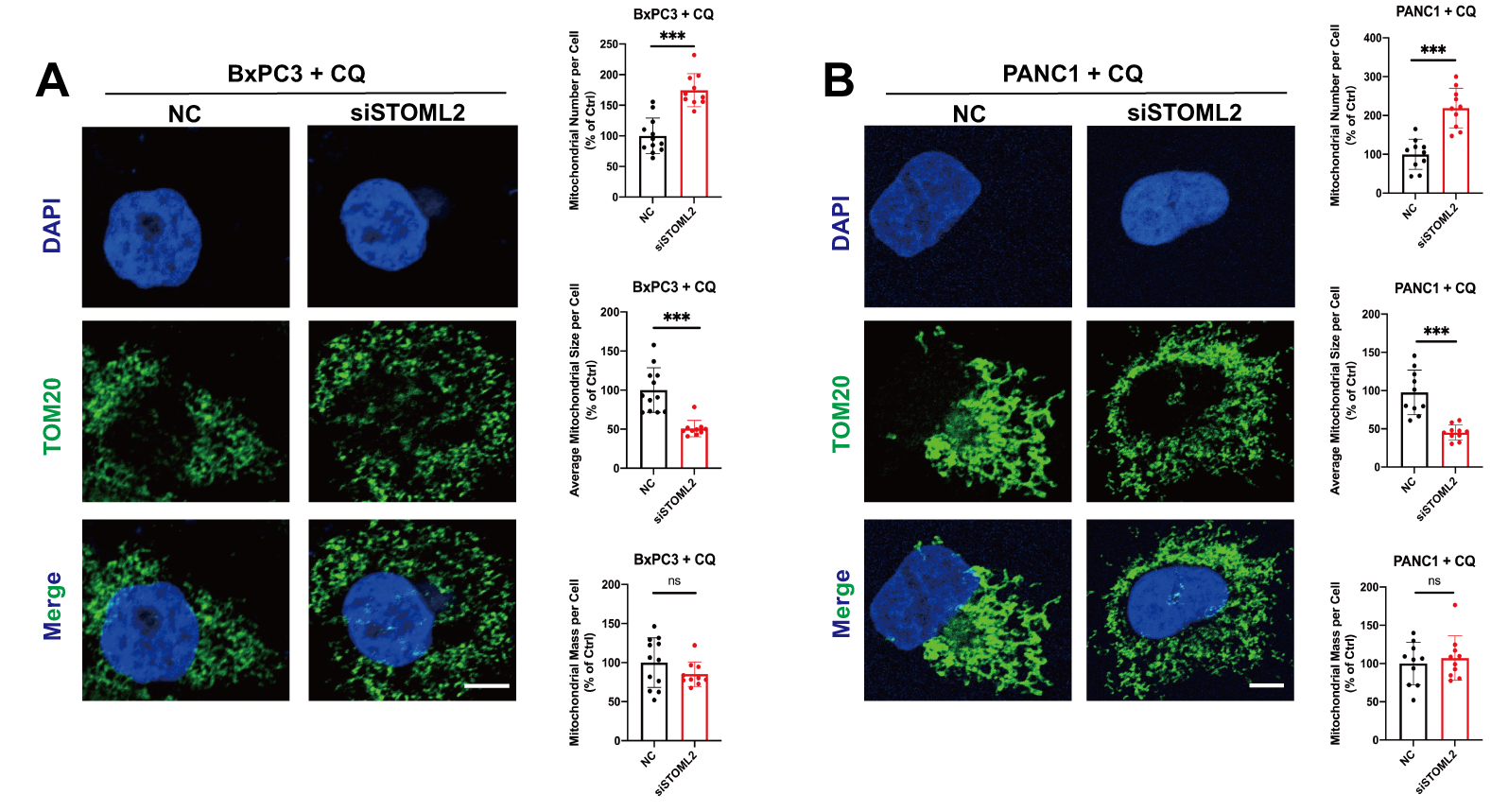

Supplement: Supplementary file 2 — Supplemental Figure 1 [file 41419_2023_5711_MOESM2_ESM.tif]

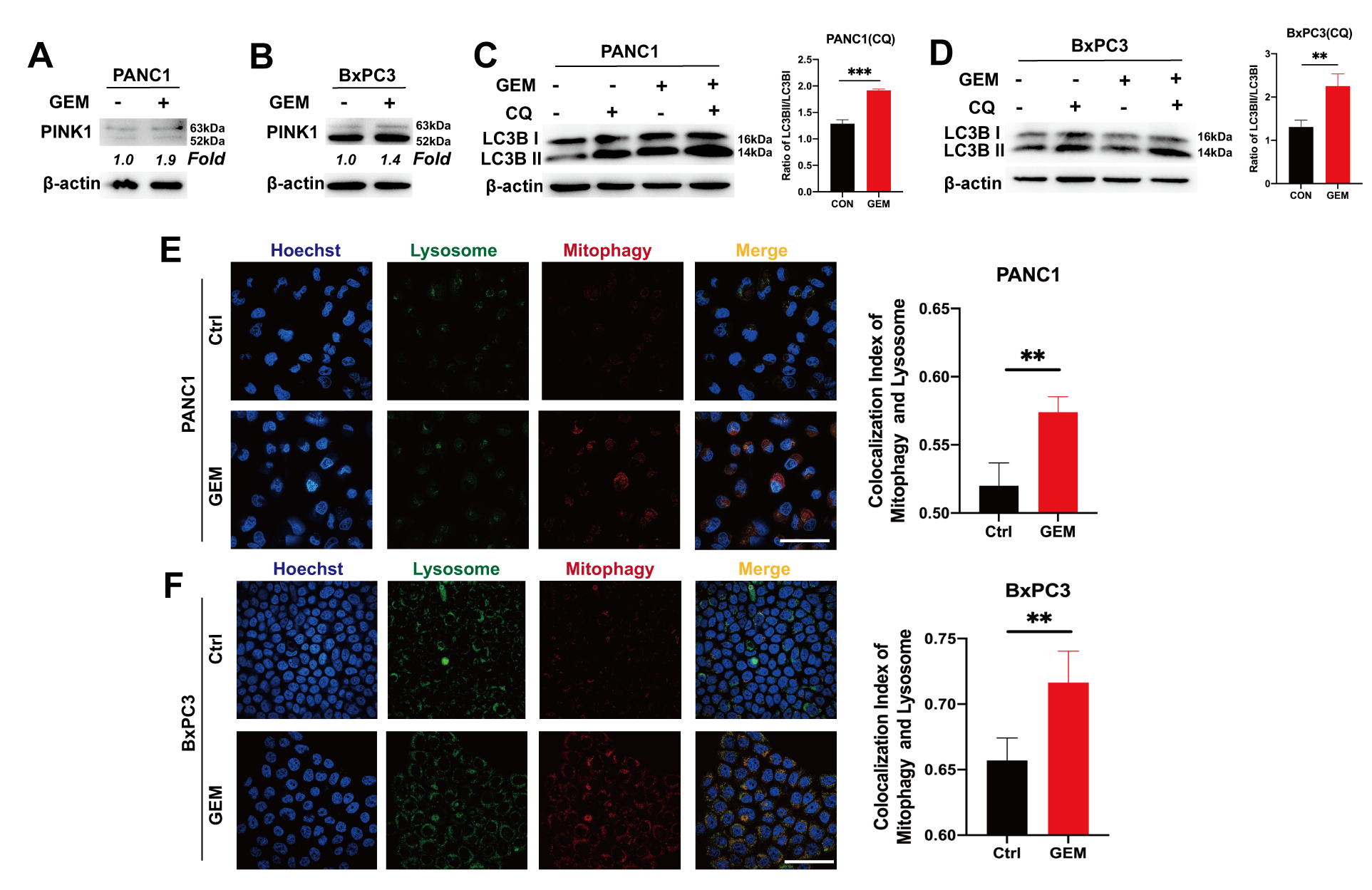

Supplement: Supplementary file 3 — Supplemental Figure 2 [file 41419_2023_5711_MOESM3_ESM.tif]

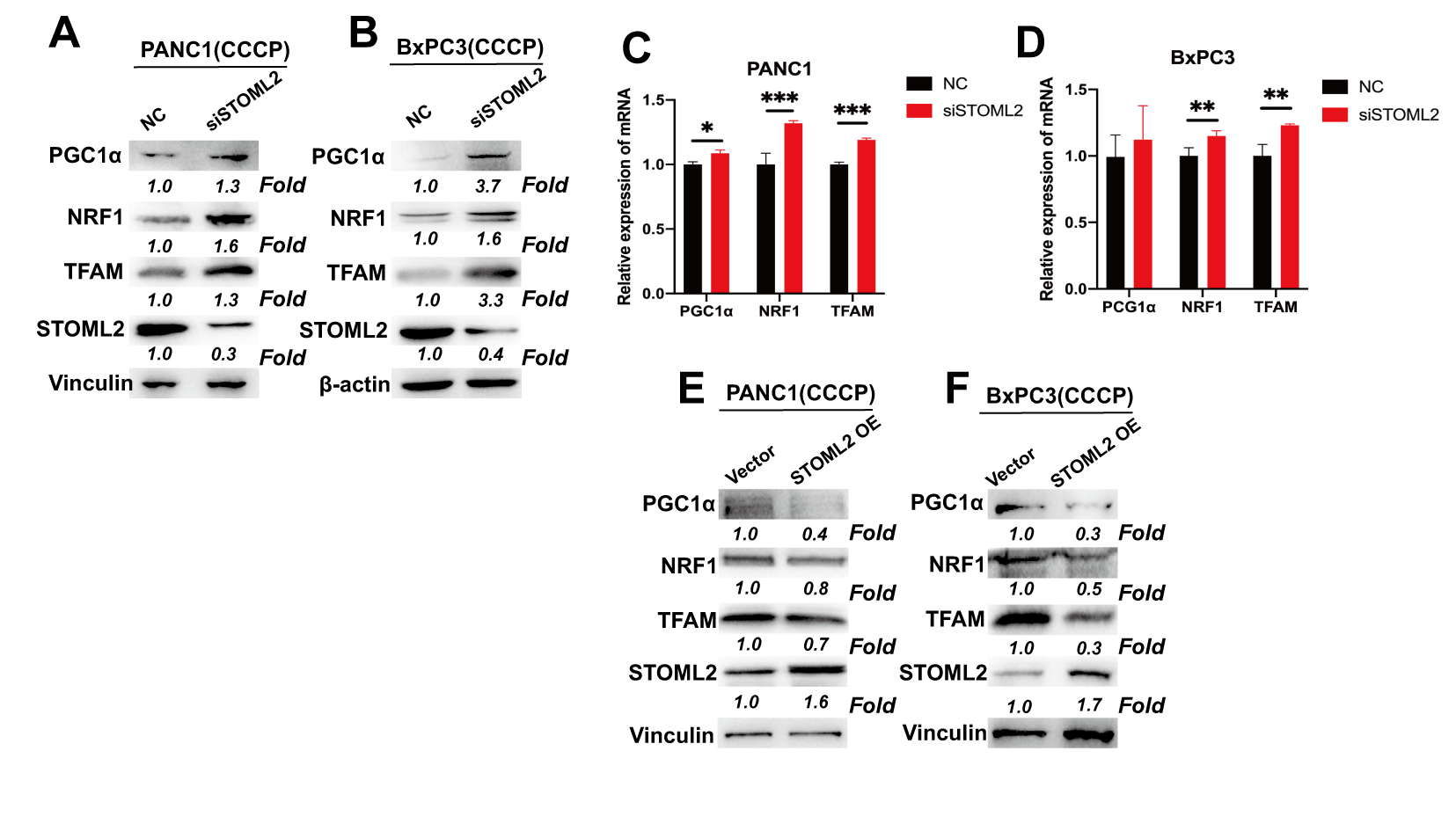

Supplement: Supplementary file 4 — Supplemental Figure 3 [file 41419_2023_5711_MOESM4_ESM.tif]

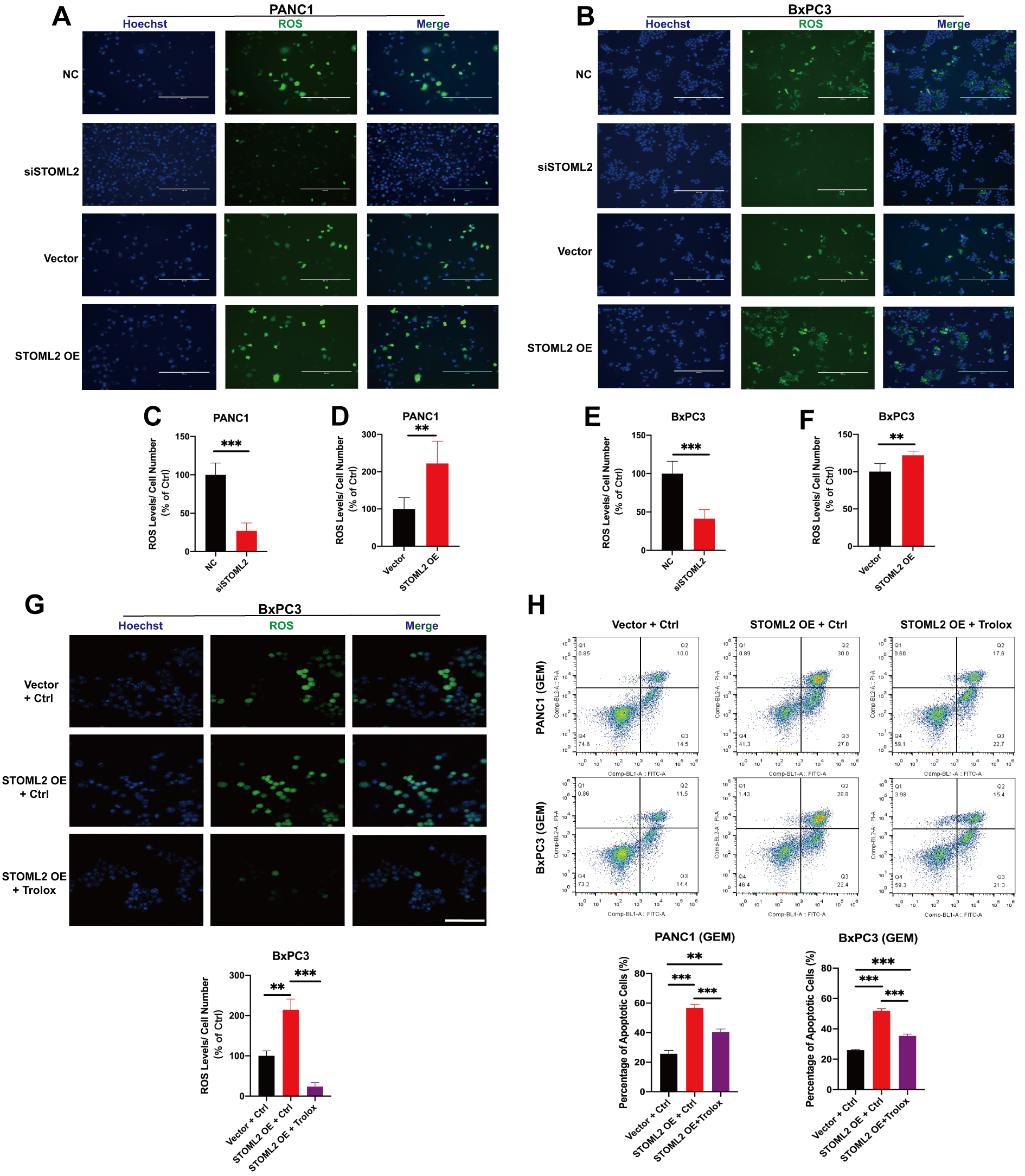

Supplement: Supplementary file 5 — Supplemental Figure 4 [file 41419_2023_5711_MOESM5_ESM.tif]
