# Supplementary material for: STOML2 restricts mitophagy and increases chemosensitivity in pancreatic cancer through stabilizing PARL-induced PINK1 degradation
Source: Cell Death Dis. 2023 Mar 11;14(3):191. doi: 10.1038/s41419-023-05711-5 (PMC10008575; doi:10.1038/s41419-023-05711-5)
Supplement: Supplementary file 7 — Supplementary Figure legends [file 41419_2023_5711_MOESM7_ESM.docx]

**Supplementary Figure 1. lysosomal inhibitor reversed the mitochondrial mass reduction induced by STOML2 downregulation.** (A-B) Immunofluorescence staining of mitochondria in BxPC3 and PANC1 cells using anti-TOM20 after STOML2 knockdown for 48 h and CQ treatment (10 μM) for 24 h. The statistical analysis of mitochondrial number, size, and mass employed over 10 random fields in each group. Scale bars equal 5 μm. ***: P < 0.001.

**Supplementary Figure 2. GEM promoted PINK1-induced mitophagy in pancreatic cancer cells.** (A-B) Immunoblot showing the protein levels of PINK1 and β-actin in PANC1 and BxPC3 cells after treatment with GEM (10 μM for PANC1 and 1 μM for BxPC3) or control in cell medium for 24 h. (C-D) Immunoblot showing the protein levels of LC3BI, LC3BII, and β-actin in PANC1 and BxPC3 cells treated with GEM, CQ (10 μM) or control. The corresponding ratios of LC3BII/LC3BI were calculated by the OD value of the corresponding blot band determined by ImageJ. (E) Fluorescent staining showed the colocalization between lysosomes and mitophagy (mitochondria) in PANC1 and BxPC3 cells after treatment with GEM or control. Scale bars equal 50 μm. The corresponding quantitative results were calculated by CellProfiler V4.2.1. *: P < 0.05; **: P<0.01.

**Supplementary Figure 3. STOML2 repressed mitochondrial biogenesis in pancreatic cancer cells.** (A-B) Immunoblot showing the protein levels of PGC1α, NRF1, TFAM, STOML2, and Vinculin in PANC1 and BxPC3 cells after downregulating STOML2, which were treated with CCCP for 24 h. (C-D) qRT‒PCR showed the mRNA levels of PGC1α, NRF1 and TFAM compared to β-actin in PANC1 and BxPC3 cells after downregulating STOML2. (E-F) Immunoblot showing the protein levels of PGC1α, NRF1, TFAM, STOML2 and Vinculin in PANC1 and BxPC3 cells after upregulating STOML2, which were treated with CCCP for 24 h. *: P < 0.05; **: P < 0.01; ***: P < 0.001.

**Supplementary Figure 4. STOML2 increased ROS levels in pancreatic cancer cells and promoted apoptosis.** (A-B) Fluorescent staining showed the ROS levels in PANC1 and BxPC3 cells after downregulating or upregulating STOML2. Scale bars equal 400 μm. (C-F) Corresponding quantitative results of the ratios of ROS levels/cell number were calculated by ImageJ. (G) Fluorescent staining showed ROS levels after upregulating STOML2 for 48 h and Trolox treatment (100 μM) for 24 h (STOML2 OE + Trolox) compared to the control groups. Scale bar equals 100 μm. (H) Dot plot from the flow cytometry staining and corresponding quantitative data were employed to assess the apoptotic cells of PANC1 and BxPC3 after upregulating STOML2 for 48 h and Trolox treatment (100 μM) for 24 h (STOML2 OE + Trolox) compared to the control groups, which were treated with GEM (100 μM for PANC1 and 10 μM for BxPC3) in cell medium for 24 h. *: P < 0.05; **: P < 0.01; ***: P < 0.001.
